# A Step-by-Step Guide for Geometric Morphometrics of Floral Symmetry

**DOI:** 10.3389/fpls.2018.01433

**Published:** 2018-10-10

**Authors:** Yoland Savriama

**Affiliations:** Institute of Biotechnology, University of Helsinki, Helsinki, Finland

**Keywords:** flowers, zygomorphy, disymmetry, actinomorphy, asymmetry, Procrustes fit, geometric morphometrics, Principal Component Analysis

## Abstract

This paper provides a step-by-step guide for the morphological analysis of corolla and the decomposition of corolla shape variation into its symmetric and asymmetric components. The shape and symmetric organisation of corolla are key traits in the developmental and evolutionary biology of flowering plants. The various spatial layout of petals can exhibit bilateral symmetry, rotational symmetry or more complex combination of symmetry types. Here, I describe a general landmark-based geometric morphometric framework for the full statistical shape analysis of corolla and exemplify its use with four fully worked out case studies including tissue treatment, imaging, landmark data collection, file formatting, and statistical analyses: (i) bilateral symmetry (*Fedia graciliflora*), (ii) two perpendicular axes of bilateral symmetry (*Erysimum mediohispanicum*), (iii) rotational symmetry (*Vinca minor*), and (iv) combined bilateral and rotational symmetry (*Trillium undulatum*). The necessary tools for such analyses are not implemented in standard morphometric software and they are therefore provided here as functions running in the R environment. Principal Component Analysis is used to separate symmetric and asymmetric components of variation, respectively, quantifying variation among and within individuals. For bilaterally symmetric flowers, only one component of left–right asymmetric variation is extracted, while flowers with more complex symmetric layout have components of asymmetric variation associated with each symmetry operator implied (e.g., left–right asymmetry and adaxial–abaxial asymmetry). Fundamental information on the genetic, developmental, and environmental determinants of shape variation can be inferred from this decomposition (e.g., directional asymmetry, fluctuating asymmetry) and further exploited to document patterns of canalization, developmental stability, developmental modularity and morphological integration. Even if symmetry and asymmetry are not the primary interest of a study on corolla shape variation, statistical and anatomical arguments support the use of the framework advocated. This didactic protocol will help both morphometricians and non-morphometricians to further understand the role of symmetry in the development, variation and adaptive evolution of flowers.

## Introduction

Symmetry has long been recognised as a key feature of the anatomical organisation and shape layout of the corolla of flowers. It is involved in numerous facets of the adaptive evolution of flowers, from plant–pollinator interaction, shifts from outcrossing to selfing, to plasticity, and response to biotic and abiotic stresses (Endress, 2001; Rodríguez et al., 2004; Sargent, 2004; Shipunov and Bateman, 2005; Bateman and Rudall, 2006; Gómez et al., 2006; Busch and Zachgo, 2007; Nattero et al., 2010; Frey and Bukoski, 2014; Vujić et al., 2015; Wang P. et al., 2015; Carleial et al., 2017; Sauquet et al., 2017; Spencer and Kim, 2018). Developmental genetic studies have started to unravel the origins of floral symmetry and identified specific genes known to play a role in the establishment of symmetry in corolla shape and related structures (Almeida et al., 1997; Luo et al., 1999; Citerne et al., 2010; Berger et al., 2017; Spencer and Kim, 2018). For instance, repeated gene duplication events are responsible for the complex ontogenetic patterning of the capitulum inflorescence, a structure thought to play a major role in the evolutionary diversification of angiosperms (Asteraceae and Dipsacaceae, Dipsacales) (Carlson et al., 2011; Berger et al., 2016). In such systems, flowers are tightly arranged according to an overall spiral symmetry (Fibonacci sequence) while the flowers themselves have symmetries varying from bilateral symmetry (zygomorphy), left–right asymmetry, to rotational symmetry depending on their position relative to the centre of the capitulum (e.g., Carlson et al., 2011; Berger et al., 2016).

Despite the ubiquity of floral symmetry and its widely acknowledged role in the evolutionary dynamics of flowers, very few studies have supplied quantitative characterisations of floral shapes and of their patterns of symmetric organisation. This mainly results from the still limited use of morphometric approaches in ecological and developmental studies of floral variation and also from the lack of user-friendly software for the statistical analysis of complex symmetry in biological shapes.

Geometric morphometrics is a collection of approaches that provide a mathematical description of biological forms according to geometric definitions of their size and shape. It is demonstrated below how these approaches can be used to precisely study corolla shape and dissect its pattern of symmetric organisation (Herrera, 1993; Gómez et al., 2006; Frey et al., 2007; Savriama et al., 2012; Hernández-Ramírez and Aké-Castillo, 2014; Wang C.N. et al., 2015; Radović et al., 2017; Strelin et al., 2018; Tucić et al., 2018). A recent landmark-based geometric morphometric framework for the complete analysis of any type of symmetry in 2D or 3D has been proposed to address in greater detail the biological significance of floral symmetry (Savriama et al., 2012). This general approach is able to unambiguously separate different components of variation as variation among flowers (symmetric component) and variation within flowers (asymmetric variation, that is the variation among the different parts composing the flower).

Here, I provide complete and detailed step-by-step protocols for the morphometric analysis of corolla shape variation, from the data acquisition to the statistical shape analysis of symmetry and asymmetry in corolla shape. All the morphometric treatment is done with freely available software (TPS Dig2, ImageJ, R). I illustrate these methods with four fully worked out examples based on previously published data as well as simulated data: bilateral symmetry (zygomorphy) in *Fedia graciliflora* (Valerianaceae), two perpendicular axes of bilateral symmetry (bi- or disymmetry) in the crucifer *Erysimum mediohispanicum* (Brassicaceae), rotational symmetry only in the pinwheel *Vinca minor* (Apocynaceae), and bilateral symmetry combined with rotational symmetry in *Trillium undulatum* (Melanthiaceae). The mathematical background underlying this morphometric framework is also briefly reviewed and three R functions are provided to address the lack of available software and make the framework accessible and applicable to any instance of corolla shape organisation.

## Background

### Geometric Morphometric Methods

In this paper, I focus on landmark-based geometric morphometric methods (GMMs) to analyse shape and size of flowers. Landmarks are Cartesian coordinates of points in 2D or 3D that can be localised precisely and without ambiguity on a structure and from one specimen to another. For instance, the points at the intersection between primary and secondary veins or at the connexions between these veins and the petal boundary are suitable landmarks in flowers (Gómez et al., 2006; Gómez and Perfectti, 2010; Savriama et al., 2012). Some landmarks are clearly defined on a structure and are named Type I (e.g., intersection between veins), others that are more ambiguous and usually describe maxima of curvature are called Type II (e.g., petal lobe), and those that are geometric constructions generated from lines or else are labelled Type III (Slice et al., 1996; Bookstein, 1997). Other data points named semilandmarks require a specific mathematical treatment and are free to slide to capture the geometry of curves and surfaces where landmarks cannot be identified such as on smooth objects (Bookstein, 1997; Gunz et al., 2005; Gunz and Mitteroecker, 2013). In this study, I only focus on landmarks to simplify the workflow. Note that the methods explained in this paper are also applicable to semilandmarks.

Once landmark configurations have been acquired on sets of digital images or on 3D objects, a Generalised Procrustes Analysis (GPA) is performed on landmark configurations and consists in minimising the sum of squared distances between corresponding landmarks to extract shape data by removing the extraneous information of size, location and orientation. A mean shape configuration (consensus) is calculated and variation around this mean can be decomposed into components of morphological variation. Shape spaces are curved and a projection onto a tangent space with the consensus as the point of tangency is used to create a shape tangent space (similarly to the projection of the earth onto a 2D map). In this shape tangent space, conventional Euclidean statistical methods are viable, such as Principal Component Analysis (PCA). Centroid size, the most common and explicit measure of size in geometric morphometrics, is computed as the square root of the sum of the squared distances of all landmarks from their centroid (Rohlf and Slice, 1990; Goodall, 1991; Slice et al., 1996; Dryden and Mardia, 1998).

### Visualisation and Quantification of Morphological Variation With Principal Component Analysis

Principal Component Analysis is a well-established method routinely used to visualise the general patterns of morphological variation in multidimensional data obtained with GMMs. PCA is the eigenanalysis of the covariance matrix of the Procrustes coordinates obtained after GPA of the original landmark coordinates. A linear combination of an eigenvector of the covariance matrix or sum of squares and cross products matrix from the original variables is calculated and produces principal components (PCs). The eigenvalues associated with each PC represent their variance. The PCs that are produced are a set of fewer and uncorrelated linear combinations of the variables from the original larger dataset. Thus, PCA reduces the dimensionality of a dataset and allows for an easier graphical representation of multivariate data. The first PC always explains the maximum possible variance, the second PC always explains the maximum possible variance after PC1, and so on for the following PCs (Jolliffe, 2002; Abdi and Williams, 2010).

### Geometric Morphometrics of Symmetry

**FIGURE 1:**
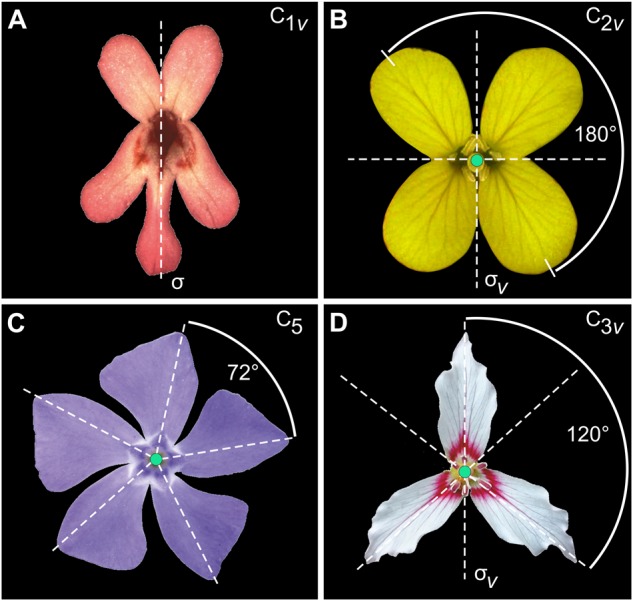
Examples of symmetry in flowers. **(A)** *Fedia graciliflora* with reflection symmetry (zygomorphy). **(B)** The crucifer *Erysimum mediohispanicum* with two perpendicular axes of reflection symmetry (dashed lines; bi- or disymmetry). Credit: photograph courtesy of J. M. Gómez, Universidad de Granada, Spain. **(C)** *Vinca minor* showing rotational symmetry of order 5 only. Symmetry groups are indicated according to the Schoenflies notation as well as the relevant symmetry operators in each case (σ: reflection line or plane of bilateral symmetry; σ_v_: vertical reflection line or plane of bilateral symmetry). Credit: photograph by Beentree, distributed under a Creative Commons Attribution-Share Alike 3.0 Unported licence. **(D)** The painted trillium *Trillium undulatum* that shows reflection symmetry combined with rotational symmetry of order 3 or actinomorphy (the axis of rotation is shown by the black dot at the centre of the flower and the angle of rotation is represented by the curved arrow). Credit: photograph by Nicholas A. Tonelli, distributed under a cc-by-2.0 licence.

Flowers and many other botanical systems often exhibit symmetric patterning in the organisation of their anatomical parts (**Figure 1**). A general approach for organisms with any type of symmetry has been designed (Savriama and Klingenberg, 2011). In this section, I present a brief, but technical overview of this framework which means that the reader unfamiliar with the concept of symmetry rooted in a mathematical context might find it rather difficult to follow. Instead, the reader could directly go to the Section “Protocols for Imaging and Collecting Landmark Data on Flowers” for imaging protocols and data acquisition, and select the case study of interest among the examples presented in the Section “Dataset Preparation and Statistical Shape Analysis of Bilateral Symmetry (Zygomorphy), Two Perpendicular Axes of Symmetry (Bi- or Disymmetry), Rotational Symmetry Only, and Bilateral Symmetry Combined With Rotational Symmetry (Actinomorphy) in Flowers.”

#### Definition of Symmetry

The symmetry of an object is defined as its invariance to one or more geometric transformations applied to it. These particular transformations are called symmetry transformations and together define the symmetry of the object (Weyl, 1952; Rosen, 1975; Flurry, 1980; Martin, 2012; Armstrong, 2013; Conway et al., 2016). For example, reflection about a flower’s mirror axis (or plane) is the symmetry transformation that leaves the flower invariant and characterises bilateral symmetry in the snapdragon *Antirrhinum majus* or *F. graciliflora* (**Figure 1A**). *E. mediohispanicum* flowers are symmetric regarding two perpendicular axes of bilateral symmetry, a vertical left–right axis and a horizontal one that separates a superior compartment (adaxial) from a lower one (abaxial) (**Figure 1B**), while others only possess rotational symmetry (**Figure 1C**) or show a combination of reflection and rotational symmetry (**Figure 1D**).

#### Types of Symmetry in Flowers

Bilateral symmetry is the simplest type of symmetry in biological systems and for this reason it has been extensively studied notably in animal groups (Leamy, 1984; Palmer and Strobeck, 1986; Møller and Swaddle, 1997; Klingenberg and McIntyre, 1998; Polak, 2003; Hallgrimsson et al., 2015). However, the diversity of symmetry types in plants is much greater. The organisation of petals in flowers can exhibit rotational symmetry only (e.g., *Vinca minor*, **Figure 1C**), combinations of reflection symmetry with rotational symmetry, and scaling symmetry also called spiral or helical symmetry in structures that approximate Fibonacci numbers (i.e., phyllotaxis, e.g., *Scabiosa columbaria* and *Knautia arvensis*, Dipsacales) (Backlund and Donoghue, 1996). These primary types of symmetry are often combined in flowers and produce more complex types of symmetry in two or three dimensions (e.g., *Trillium undulatum*, **Figure 1D**).

**FIGURE 2:**
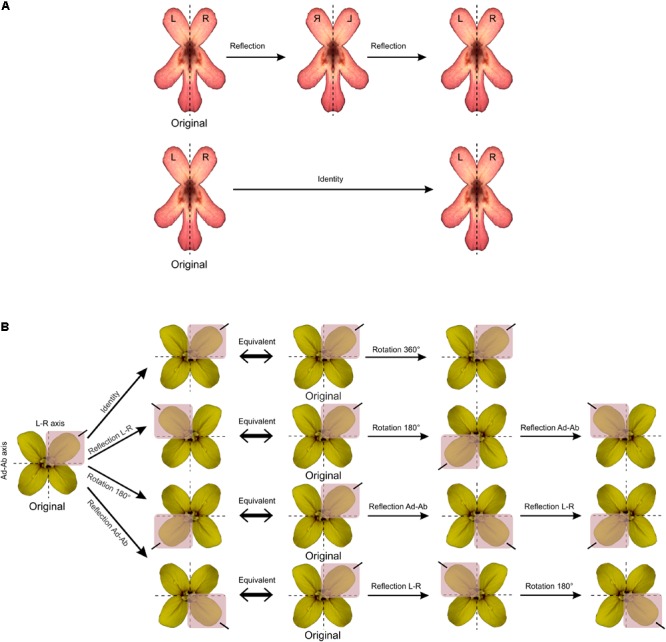
The concept of symmetry group in mathematics. **(A)** *F. graciliflora* flowers are symmetric with respect to a set of two symmetry transformations: the identity (i.e., the transformation that does nothing) and reflection about a vertical axis. This set defines the symmetry group of *Fedia* that is bilateral symmetry or zygomorphy. **(B)** *E. mediohispanicum* flowers are symmetric regarding two perpendicular axes of symmetry and to a set of four symmetry transformations: the identity, reflection about the vertical left–right axis, reflection about the horizontal adaxial–abaxial axis, and rotation by 180°. These symmetry transformations are mutually equivalent when combined with each other.

Each type of symmetry is related to a set (in the mathematics sense) of symmetry transformations. For instance, bilateral symmetry is defined by a set of two symmetry transformations: reflection about the mirror axis and the identity. The identity is the symmetry transformation that does nothing and is defined as such since it is a key element in the mathematical concept of symmetry (Rosen, 1975; Flurry, 1980). For instance, applying two successive reflections about the same mirror axis or rotations by 360° are also equivalent to applying no transformation at all (**Figure 2A**). The set of all symmetry transformations of an object defines a mathematical group named the symmetry group of the object (Martin, 2012; Armstrong, 2013; Conway et al., 2016). The framework of group theory have been applied for the study of symmetry. While some symmetry groups are finite with a countable number of symmetry transformations, others are infinite since they comprise symmetry transformations that are coupled with translations (Rosen, 1975; Flurry, 1980). For example, the symmetry group of *E. mediohispanicum* is finite since it contains the identity, a reflection about the vertical left–right axis, a reflection about the horizontal adaxial–abaxial axis, and two successive reflections about the two perpendicular axes of bilateral (or reflection) symmetry also equivalent to rotation by 180° (**Figure 2B**).

The Schoenflies notation, which I will use throughout this study, is commonly used to define types of symmetry in crystallography (Burzlaff and Zimmermann, 2006). Most types of symmetry found in flowers are described by the following symmetry elements: *C_n_* for *n*-fold rotation axis and σ for reflection about an axis or plane:

– *n*-fold rotation axis (*C_n_*): A flower is symmetric relative to rotation about an axis by a specific angle (or/and multiples of it) so that the initial and final positions of the flower petals are indistinguishable. For example, *V. minor* is symmetric by rotation of 72°, but it is also symmetric if the same rotation is applied twice (144°), three times (216°), four times (288°) and five times (360°) (**Figure 1C**). Consequently, this flower has a fivefold rotation axis noted C_5_ according to the Schoenflies system.
– reflection axis or plane (*σ*): A flower is symmetric under reflection about an axis (or plane) if the two sides are mirror images of each other relative to this axis. Reflection about a vertical axis is denoted C_1_*_v_* and reflection across an horizontal axis is denoted C_1_*_h_*.

According to this notation, the symmetry group of bilateral symmetry (zygomorphy) in *F. graciliflora* is C_1_*_v_*, bi- or disymmetry in *E. mediohispanicum* is C_2_*_v_*, rotational symmetry only in *V. minor* is C_5_, and combination of reflection and rotational symmetry in *T. undulatum* is C_3_*_v_* [see Table 1 in Savriama and Klingenberg (2011) for a complete enumeration of finite symmetry groups]. In geometric morphometrics, two approaches have been distinguished for the study of symmetric structures: matching symmetry and object symmetry.

#### Flowers With Symmetric, but Physically Disconnected Petals (Matching Symmetry)

Matching symmetry refers to the case where a complete structure is composed of a suite of *k* repeated units physically disconnected and arranged according to a set of symmetry transformations. For instance, the left and right petals of *Tropaeolum speciosum* (Tropaeolaceae) show bilateral matching symmetry with respect to reflection about the axis (or plane) of the flower, other examples of matching symmetry can be found in the Mexican primrose-willow (*Ludwigia octovalvis*, Onagraceae), the kerria (*Kerria japonica*, Rosaceae) or “Pride of De Kaap” (*Bauhinia galpinii*, Fabaceae) (Lindley, 1836; Raven, 1977; Rix, 2010; Azani et al., 2017).

**FIGURE 3:**
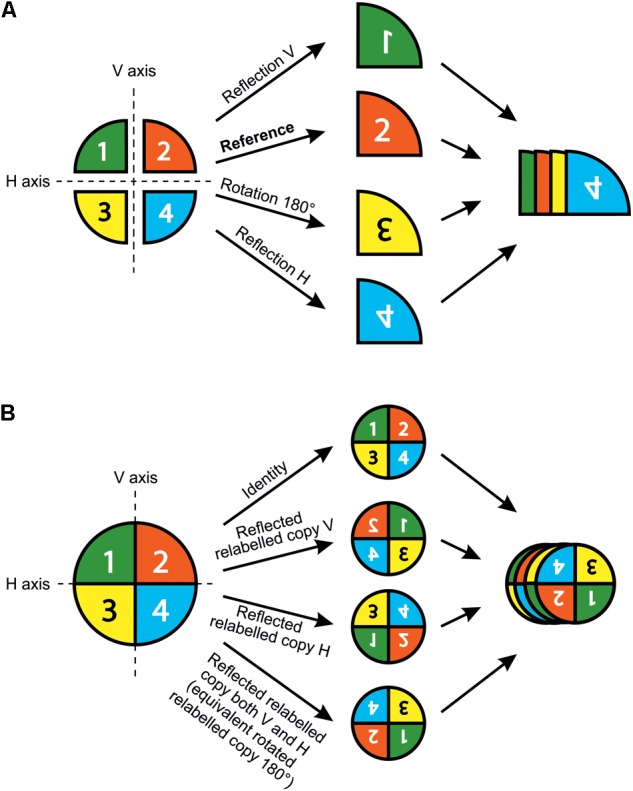
Matching and object symmetry for shape analysis of flowers with any type of symmetry. **(A)** Analysis of a flower with matching symmetry. First, the whole flower is divided into individual repeated parts or modules that generate the symmetry. Second, an individual configuration of landmarks is considered for each repeated part. A configuration is selected as a reference and reflections are applied to the rest of the images as needed so that all subsequently digitised configurations from the transformed pictures match the orientation of the chosen reference. Note that rotations are not needed here since the GPA automatically carries this step during the superimposition procedure. Finally, all individual configurations are superimposed simultaneously in a single GPA. **(B)** Analysis of a flower with object symmetry. First, an original configuration of landmarks is digitised for the whole flower. Second, *n* copies of this original configuration are generated and each of them is transformed according to one of the symmetry transformations in the symmetry group of the flower (here, *n* = 4). Finally, all original configurations and their transformed relabelled copies are superimposed simultaneously in a single GPA. The resulting mean shape (consensus) is symmetric.

The shape analysis of matching symmetry proceeds as follows: First, a separate configuration of landmarks is considered for each repeated unit. Then, one of the configurations of landmarks is selected as a reference and all the others are matched onto it by using the transformations of the symmetry group. For instance, all left petals of *T. speciosum* can be reflected to match their right counterparts by simply multiplying one of their landmark coordinates (e.g., *x*) by -1. Finally, a GPA superimposes all configurations and produces a mean shape (consensus). It is important to note that for flowers with rotational symmetry only, there is no need to manually transform the configurations before the GPA. Indeed, the relabelling is only required since the transformation needed to match the copies of the original configuration, that is the rotation, is already incorporated in the GPA. In other words, copies relabelled according to rotation will automatically be rotated to ensure label correspondence during the superimposition procedure included in the GPA (**Figure 3A**).

A component of symmetric variation that represents the variation among individuals is computed by calculating the differences among the averages of all repeated parts for all individuals. Several components of asymmetry might occur depending on the order of symmetry under study. A separate configuration of landmarks is used for each side, which means that separate values of centroid sizes can be calculated per repeated part and consequently a component of symmetric variation and asymmetry can be calculated for size as well (Klingenberg and McIntyre, 1998; Mardia et al., 2000; Klingenberg et al., 2002; Savriama and Klingenberg, 2011). Matching symmetry is suitable for the analysis of any type of symmetry even with infinite symmetry group such as translational (a constant shift of body parts along an axis, such as the arrangement of leaves along the shoot of plants), spiral or helicoidal symmetry (rotational symmetry combined with translational symmetry in which the architecture/growth of the organism follows a constant or increasing deviation from a centre or axis of rotation, such as in many Asteraceae) (Savriama and Klingenberg, 2011).

#### Flowers With Symmetric, but Physically Connected Petals (Object Symmetry)

Object symmetry describes the case of a symmetric structure for which the symmetry operators (centre, axis, or plane) belong to the structure itself and partition it into *k* physically connected compartments. The snapdragon *A. majus* is an example of bilateral object symmetry in which the plane of bilateral symmetry passes through the middle of the entire flower separating it into left and right connected halves. The analysis of structures with object symmetry takes into account the variation between connected parts as in matching symmetry, with additional information made available about the way the two halves are physically connected to each other (Mardia et al., 2000; Klingenberg et al., 2002; Savriama and Klingenberg, 2011).

For the analysis of object symmetry, a unique configuration of landmarks is considered for the entire structure, and then *n* copies of this configuration are created, where *n* is the number of transformations included in the symmetry group of the structure (**Figure 3B**). An appropriate relabelling of landmarks is necessary for each transformed copy and simply consists in mutually swapping the labels of the landmarks that are images of each other with respect to a given symmetry transformation, and this does not affect the landmarks that are placed onto the symmetry operators since they will be mapped onto themselves. A reflection combined with a number of rotations corresponding to the highest order of rotational symmetry is sufficient to generate the required number of symmetry transformations in any finite symmetry group. The full dataset is superimposed by a GPA and the resulting consensus is symmetric since all transformed and appropriately relabelled configurations have been included. Since there is a unique configuration for the whole structure and due to constraints imposed by the Procrustes fit, there is no asymmetry in size as opposed to matching symmetry. Note that object symmetry is only applicable to structures with a finite symmetry group since it requires a full enumeration of the symmetry transformations and is therefore not applicable to structures with translational symmetry. For instance, the symmetry group of *A. majus* flowers contains two symmetry transformations: reflection about the left–right axis, and the identity. Therefore, two transformed copies are included in this dataset: the identity (i.e., the original configuration of landmarks) and a reflected copy of this original configuration. The reflected copy can be easily obtained by multiplying one of its landmark coordinates (e.g., *x*) by -1.

A component of symmetric variation that corresponds to the variation among individuals is calculated as the difference among averages of all original and their transformed relabelled copies. As in complex matching symmetry, multiple components of asymmetry can occur that can be calculated as the difference between the original configuration and the transformed relabelled copies with respect to a given symmetry operator. For instance, in *E. mediohispanicum* flowers a component of asymmetry can be quantified between the adaxial and abaxial units by calculating the difference between the original configuration and the reflected relabelled copy about the adaxial–abaxial axis, but also between left and right petals by calculating the difference between the original configuration and its reflected relabelled copy about the vertical left–right axis, and across diagonally opposed petals by calculating the difference between the original configuration and the copy generated by two successive reflections about both axes (also equivalent to a rotated copy by 180°).

##### Flowers with object symmetry have a special mathematical property

The treatment of landmarks for flowers with object symmetry has particularities such as a structuration of the total shape tangent space into separate and orthogonal, but complementary subspaces of shape variation, and this has important implications for statistical tests (Kent and Mardia, 2001; Klingenberg et al., 2002; Kolamunnage and Kent, 2003, 2005; Savriama and Klingenberg, 2011). For instance, flowers with bilateral object symmetry such as the snapdragon *A. majus* have two subspaces of shape variation, a subspace of completely symmetric shape variation and a subspace of totally asymmetric shape changes. Multiple subspaces can occur with more complex types of symmetry.

A PCA applied on a dataset that contains all original and transformed relabelled copies effectively and easily separates symmetric and asymmetric components into distinct subspaces of shape variation. Given that each category of shape variation can be identified, it is straightforward to sum up the percentages of variance for each class of shape change and determine for how much they account relative to the total shape variation.

For flowers with rotations of order beyond 2 (by 180°), the PCA produces pairs of PCs with equal eigenvalues. This means there is no unique solution for the orientation of the pairs of eigenvectors associated with the eigenvalues. Consequently, shape changes expressed in this plane can correspond to a combination of different types of symmetry (e.g., absence of symmetry and bilateral symmetry; Savriama and Klingenberg, 2011) whose their respective shape patterns might not be recognisable. To overcome this difficulty, Savriama and Klingenberg (2011) recommended rotating the PCs of these pairs so that the overall mean and the mean score of all unrotated copies coincides with one of the PCs of the given pair. As a result, the shape changes related to these pairs of PCs become apparent.

Interesting complexity and further structuring in the data arise for flowers with rotational symmetry that is specifically related to their order of rotation (further technical details are covered in Savriama and Klingenberg, 2011):

– If the order of rotation is a prime number (e.g., 3 or 5), the shape tangent space is decomposed into only two kinds of orthogonal subspaces: one subspace of symmetric shape changes relative to the rotations and one subspace of totally asymmetric shape changes. This is the case for pinwheels such as *V. minor*.
– If the order of rotation is not a prime number (i.e., 6), there are several subspaces of symmetric shape changes with respect to rotations, which orders correspond to each prime factor of the original order of rotation (e.g., 2 and 3 for an order of rotation of 6). This is the case of the ‘Kleim’s Hardy’ variety of *Gardenia jasminoides* (Rubiaceae).
– If the symmetry group contains both reflection and rotations, the shape tangent space is decomposed into various subspaces as a consequence of the combinations between the reflection and rotations. This is the case of *T. undulatum*.

##### Analyses of flowers with object symmetry leads to complex structures in PCA scatterplots

With object symmetry, when one PC is plotted against another one, the resulting scatterplot shows a distribution of individuals which itself can exhibit asymmetric or symmetric patterns. This is due to the copies generated from the original dataset according to the symmetry group of the flower under study. For instance, in the case of bilateral object symmetry individual PCs can show shape changes that are either symmetric or asymmetric. If two PCs both represent shape changes that are symmetric with respect to the symmetry axis, this means that the variation displayed is inter-individual variation and that original and reflected relabelled copies have the same position on this 2D scatterplot (identical PC scores for this pair of PCs); therefore the scatterplot of PC scores will be totally asymmetric.

Principal components associated with non-symmetric shape changes imply that the projection of the data in the 2D plane defined by these PCs reflects in fact the symmetry transformations applied to the copies of the original dataset. For instance, PCs representing totally asymmetric shape changes for a dataset describing flowers with threefold rotational symmetry (i.e., by 120°) will show PC scores that actually represent the original dataset and its transformed copies. Consequently, these 3 replicates will be oriented following a rotational symmetry by 120° layout. Conversely, if PCs have shape changes that are related to the transformations included in the symmetry group of flower that has rotational symmetry, then the showcased variation will be inter-individual variation and the 2D scatterplot of PC scores will be asymmetric. These phenomena extend to more intricate types of symmetry and in these cases scatterplots of PC scores can exhibit more complex symmetric patterns (Kent and Mardia, 2001; Kolamunnage and Kent, 2003; Kolamunnage and Kent, 2005; Savriama et al., 2010; case study 3 and 4).

#### What if Floral Shape Exhibits Combination of Flowers That Are Symmetric, but Themselves Arranged According to Another Symmetric Pattern (Both Matching and Object Symmetry)?

In many clades, flowers often possess petals or other parts that have bilateral object symmetry and are themselves arranged according to more complex symmetric patterns. In Dipsacales (e.g., *Scabiosa columbaria, Knautia arvensis*), flowers are tightly assembled together into a structure called the capitulum and have symmetries ranging from zygomorphy, left–right asymmetry, to rotational symmetry, but are themselves arranged according to spiral symmetry that follows a Fibonacci sequence (e.g., Carlson et al., 2011; Berger et al., 2016). In the European orchids (*Dactylorhiza*), flowers within a single inflorescence are often arranged according to symmetric patterns (Bateman and Rudall, 2006). In this case, the user can decide that the repeated unit of interest is the symmetry of the flower, which will be symmetrised first (object symmetry), while the higher order of symmetry found in the arrangement of flowers according to complex matching symmetry is automatically taken into account in the rotation step incorporated in the GPA. This approach has been recently used to study translational fluctuating asymmetry and developmental costs in modular organisation of centipedes. In this case study, the bilateral object symmetry of each segment along the body axis was first taken into account and the translational matching symmetry was automatically treated when all symmetrised segments were superimposed during a single GPA (Savriama et al., 2016, 2017).

Within a flower, one can also consider that the petal is the repeated unit of interest and if it has bilateral object symmetry, then each petal can be first symmetrised before superimposing all of them in a single GPA. This was recently applied to dissect the sources of variation due to phenotypic plasticity and fluctuating asymmetry in *Iris pumila* (Iridaceae) (Tucić et al., 2018). A general approach for organisms with such composite types of symmetry arranged according to a hierarchical fashion has been recently designed (Savriama and Gerber, 2018).

## Protocols for Imaging and Collecting Landmark Data on Flowers

### Imaging Procedures

For flowers that cannot be removed from the plant and are relatively flat, high definition photographs should be taken from standardised positions according to a front view and a side view such that petals appear coplanar in either of these views (Gómez et al., 2006). All pictures should be taken with a scale next to the specimen and at the same distance from the camera.

**FIGURE 4:**
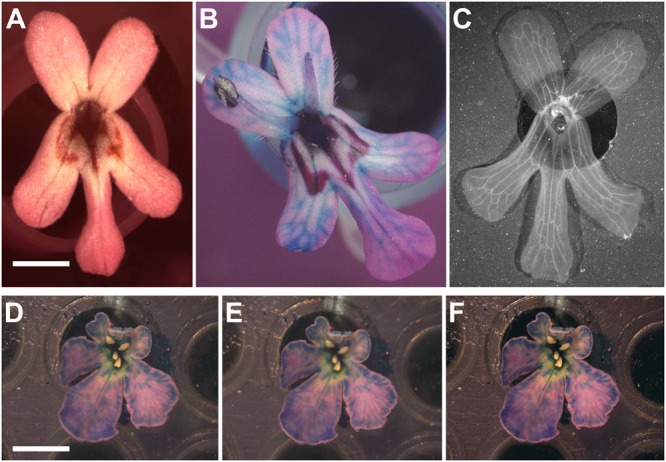
Revealing non-visible veins in corolla of flowers. **(A)** Wild-type of *F. graciliflora* flower mounted on a petri dish. **(B)** The absorption of a coloured liquid by the flower reveals its vascular system. As an example, the venation system of *F. graciliflora* is entirely revealed after the flower had spent several hours absorbing a solution made of blue dye. **(C)** *F. graciliflora* flowers that are relatively flat can be immersed into a solution of 70% ethanol to effectively destroy the cells responsible for pigmentation and reveal their venation network in high detail. Scale bar = 2 mm. **(D,E)** Clarity of pictures taken from non-2D flowers (*Scabiosa columbaria*, Dipsacaceae) can be improved via z-stacking or focus stacking that combines a set of pictures acquired at different focal points of the flower. **(F)** These images are subsequently compiled into a single and neat picture. All visible organs that could mask the corolla (e.g., stamen and carpel) should be discarded prior to any imaging. Scale bar = 5 mm.

For flowers that can be taken off the plant, the procedure consists in removing the flower while preserving its stem and discarding all visible organs that could mask the corolla (e.g., stamen and carpel). Prior to imaging, flowers should be placed at a fixed position by mounting them using an appropriate support (e.g., the wells of a PCR plate) and placing their petals facing up (**Figure 4A**). Afterward, pictures can be taken from a camera mounted on a dissecting microscope depending on the size of flowers.

Some flowers might not show their venation system and this renders landmark acquisition more difficult or nearly impossible. To circumvent this issue, one can use food colourant (e.g., blue dye) that will be absorbed via the vascular system contained in the stem of the flower and subsequently spread all over the corolla, ultimately revealing the network of veins. As an example, the venation system of *Fedia* is entirely revealed after the flower had spent several hours absorbing a solution made of blue dye (**Figure 4B**). A more radical alternative is to immerse flowers in a solution of 70% ethanol to effectively destroy the cells responsible for pigmentation subsequently fully revealing their network of veins. This approach gives the clearest results and the treatment will unambiguously highlight the veins. This is an extremely efficient and fast way to perfectly accent the details of a venation network in flowers (**Figure 4C**). Several flowers can be treated at once by first mounting them on a support (e.g., PCR plate) that is itself immersed in a pool made of 70% ethanol, with the whole system being placed inside a sealed container to limit the evaporation of the solution.

For flowers that are almost but not quite 2D flat structures taking a single picture does not prove to be sufficient since parts of the corolla will be out of focus thereby producing blurry images. To improve the clarity of pictures taken from such flowers, one can use z-stacking or focus stacking that treats a set of pictures acquired at different focal points of the flower and subsequently compiles them into a single and neat image (**Figures 4D–F**).

All pictures should be taken with the same settings (e.g., camera model, resolution, objective, distance from the specimen). If all pictures are taken with the same settings, there is no need to have a ruler on each of them, but only in one of them, which will be considered for all pictures at once.

### Measurement Error

Measurement error can occur when data are acquired for example on low resolution photographs or when pictures are taken in a non-standardised manner (Bailey and Byrnes, 1990; Arnqvist and Martensson, 1998; Fruciano, 2016). The overall measurement error in shape analysis is traditionally dissected relative to “Imaging” (i.e., differences in pictures of the same flower) and “Digitising” (i.e., differences in landmarking the same picture of a flower) in an Analysis of Variance (ANOVA) for size (centroid size) and shape (Procrustes coordinates) (Klingenberg and McIntyre, 1998; Klingenberg et al., 2002; Savriama and Klingenberg, 2011).

A one-way Procrustes ANOVA design can be used to test for measurement error directly against the biological signal of interest that is the variation among flowers. For flowers with matching symmetry, this design is readily applicable to the full dataset since the different components of shape changes (symmetric and asymmetric) belong to the same shape space. For studies of object symmetry, each component of shape change (symmetric and asymmetric) belong to separate shape spaces and each of them has different shape dimensions. Consequently, the user has to carefully determine the correct shape dimensions for each shape space in this particular context (Goodall, 1991; Klingenberg et al., 2002; Savriama and Klingenberg, 2011), and an easier alternative is to run the analysis using the original configurations only [see the Section “Case Study 1: Bilateral Object Symmetry in *F. graciliflora* (Symmetry Group C_1_*_v_*)].

### Image Preparation Prior to Landmarking

Several software exist for landmark acquisition such as ImageJ (Schneider et al., 2012) or the function ‘*digitize2d()*’ in the ‘geomorph’ R package (Adams and Otárola-Castillo, 2013). Here, I use the popular ‘*tps*’ suite of software to collect and organise files for landmark data collection since it is the simplest solution for users non-familiar with other alternatives, especially the ones that require programming (Rohlf, 2015). The two software needed are ‘tpsUtil’ and ‘tpsDig2’ (Rohlf, 2015). These software run on Windows only, but work perfectly well on Mac computers via an appropriate emulator (e.g., Parallels, WineBottler). Prior to digitising, images should be transformed depending on whether or not the flower has either matching symmetry or object symmetry as explained in the following sections.

#### Image Preparation for Flowers With Matching Symmetry

In the case of flowers with matching symmetry, it is best to include separate pictures of each repeated unit together in a unique folder. If at least two pictures of each module have been taken, measurement error due to mounting can be appraised. To do so, each picture is duplicated and landmarks should be acquired twice for each of these two copies to assess measurement error due to digitising. For instance, for flowers with bilateral matching symmetry the pictures can be named as follows:

Ind001_R_a1b1
Ind001_R_a1b2
Ind001_R_a2b1
Ind001_R_a2b2
Ind001_L_a1b1
Ind001_L_a1b2
Ind001_L_a2b1
Ind001_L_a2b2

With ‘Ind001’ denotes the specimen, ‘L’ or ‘R’ is the repeated unit (Left or Right in the case of bilateral symmetry), ‘a’ refers to the image and ‘1’ or ‘2’ represents the session, ‘b’ stands for the digitising and ‘1’ or ‘2’ refers to the session. Prior to digitising, choose a picture of one of the repeated unit as a reference and flip/rotate all pictures so that they have the same orientation as the one chosen as a reference. This reduces risks to inflate the level of artefactual directional asymmetry that could be caused by the experimenter’s handedness.

#### Image Preparation for Flowers With Object Symmetry

For flowers with object symmetry, unique pictures of the full corolla are included in the same folder. If two separate pictures of the same flower have been taken, measurement error due to mounting can be quantified. Each picture is duplicated and two series of landmarks should be acquired for each of these two copies to assess measurement error due to digitising. Consequently, the files should be named as follows:

Ind001_a1b1
Ind001_a1b2
Ind001_a2b1
Ind001_a2b2

Reading from left to right, with ‘Ind001’ denotes the specimen, ‘a’ is the image and ‘1’ or ‘2’ is the repeat for a given imaging session, ‘b’ is the digitising and ‘1’ or ‘2’ refers to the repeat. A unique configuration of landmarks encompasses the repeated units of the full flower together as opposed to analyses with matching symmetry.

### Landmarking With tpsDig2

In this section, I use *Fedia graciliflora* flowers which possess an instance of bilateral object symmetry (zygomorphy) as an example to describe the procedure for landmarking with ‘*tpsDig2*’ (see the Section “Image Preparation for Flowers With Object Symmetry” for preliminary image preparation).

^∗^First, use ‘*tpsUtil*’ to generate a file template as follows:

(1) Operations: “Build tps file from image”
(2) Input directory: click on “Input” and choose the folder that contains the pictures. Click on the first picture in the folder, then click “Open.” This brings you back to ‘*tpsUtil.*’
(3) Click on “Output” and choose the folder that contains the pictures. Type the desired file name in the “File name” tab followed by the extension ‘.TPS.’ For instance, the file can be named as “FediaProjecta1b1.TPS,” which will contain all landmark coordinates digitised for pictures taken during the first session. Click on Save. This brings you back to ‘*tpsUtil.*’
(4) Click on ‘Setup.’ This opens a new window in ‘tpsUtil’ in which all the available images can be selected/unselected.
(5) Once the user is satisfied with the list of images to be digitised, the ‘.TPS’ file can be created by clicking on “Create.” A preview of the file to be created also appears. Click “Close” to complete the procedure. Cheque the newly created ‘.TPS’ file by simply opening it with any text editor. One can make any change to this file using a text editor and such modifications will be recognised and taken into account when read back again in ‘*tpsDig2.*’ Repeat the whole procedure to create the other files corresponding to the different digitising sessions such as “FediaProjecta1b2.TPS,”

“FediaProjecta2b1.TPS,” and “FediaProjecta2b2.TPS.”

^∗^Second, Add a scale factor.

Prior to imaging, a scale should be placed next to each specimen and should appear on each photograph. If pictures have been taken using the same settings, one can apply the same scale factor to all of them. If the latter has been defined at the beginning of the digitising session, ‘tpsDig2’ automatically carries the same scale factor to all pictures.

(1) Open a picture containing a scale. Set up the factor that converts pixels in the desired units. In the tool bar, click on “Image Edit Tools,” select the “Measure” tab. In the “Scale factor” section, select the appropriate reference and unit. Then, click “set scale.” Digitise the starting and ending points of the graduation of the scale in the picture that define a given length in pixels corresponding to the unit determined earlier. For instance, choose “1 millimetre” and click “Ok” to record this conversion factor. Alternatively, there is an option in ‘tpsUtil’ to define a scale factor after all pictures have been taken. To add this scale factor afterward, open the ‘.TPS’ file with ‘tpsUtil’ and use “add variable.” Simply enter “scale=” in “variables” and the value of the scale in the value tab. This conversion factor will be applied to all specimens listed in the ‘.TPS’ file.

^∗^Third, digitising landmarks with ‘tpsDig2.’

**FIGURE 5:**
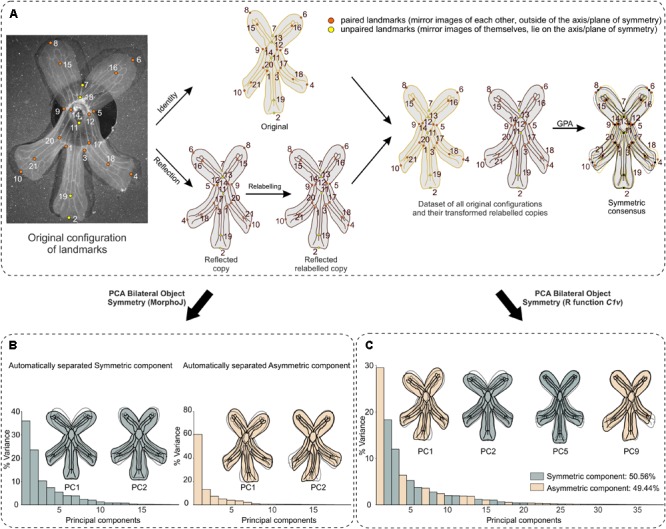
Workflow for shape analysis of bilateral object symmetry in *F. graciliflora* (case study 1). **(A)** First, an original configuration is considered, then a reflected copy is generated and relabelled with the landmarks that are mirror images of each other with respect to the axis (or plane) of symmetry are mutually swapped (paired landmarks) while the ones that lie on the symmetry axis are mapped onto themselves (unpaired landmarks). A Generalised Procrustes Analysis is applied to this doubled dataset to extract shape data (Procrustes coordinates) by removing extraneous information of size, location and orientation via a least-squares criterion. The mean shape (consensus) is symmetric. **(B)** MorphoJ’s implementation of bilateral object symmetry automatically separates a component of symmetric variation (i.e., variation among flowers) from the asymmetry (i.e., variation within flowers or between left and right sides of flowers). Consequently, separate PCAs are run on the covariance matrix of the Procrustes coordinates for each component to quantify and display their patterns of shape changes, but do not indicate how much these PCs account for the total amount of shape variation. For each component, percentages of variance for which the PCs account are reported and the first 2 PCs and displayed. The outline drawings of flowers show shape changes associated with each PC from the overall average shape (dotted outline and open circles) for PC1 and PC2 scores of +0.1 (solid black outline with gray background and solid black circles). Note that these outline drawings are an interpolated form of display from the real landmarks based on the thin-plate spline method that makes it easier to visualise shape changes. This means that the interpretable information is from the positions of the landmarks, not from the outline drawings. **(C)** A PCA applied on the same superimposed already doubled dataset obtained via function ‘*C1v*’ unambiguously separates these components of shape variation and indicates how much their associated PCs account for the total variance. The first two PCs of each component are displayed. The outline drawings are generated as in **(B)**.

Before starting digitising landmarks, the user needs to define an appropriate configuration (in 2D or 3D) that can be localised precisely and without ambiguity on structures and easily reproducible across specimens (e.g., intersection of veins). Others that are less precise, but still relevant in some cases can be placed at the maxima of curvature (e.g., petal lobe). The configuration of landmarks should provide a decent coverage of the whole flower and be well defined enough to efficiently capture the overall morphology of the flower. For instance, the configuration chosen for *F. graciliflora* uses landmarks placed at maxima of curvature of petals to describe the external overall differences among petals, but also other landmarks can be found inside the flower at intersection between veins to pick even more internally intricate patterns of variation (**Figure 5A**). A rule of thumb states that the total number of landmarks should be less than half or a third of the number of individuals (respectively, in 2D and 3D) to carry out appropriate analyses. For instance, if the user decides to collect 15 landmarks in 2D, then a little more than 30 individuals will be needed, otherwise there will be fewer individuals than variables and this could lead to statistical problems. It is crucial that all landmarks from each picture are digitised following the same order, otherwise this will lead to a spurious superimposition during the Procrustes fit (GPA).

(1) Open the “FediaProjecta1b1.TPS” file template previously created in ‘tpsUtil.’ Select ‘File, Input source’ and choose “FediaProjecta1b1.TPS.” ‘tpsDig2’ displays the first image contained in the folder that is listed/associated with the .TPS file template previously created and allows the user to navigate through the rest of them. Select ‘Options’ and activate “mouse wheel zooms” to speed up and facilitate the digitising procedure.
(2) Start digitising landmarks by clicking on the tool bar “Digitise landmarks,” the pointer of the mouse will take the appearance of a target symbol and use mouse left click to digitise landmarks. Landmarks can be edited (e.g., shifted and deleted) at any time by opening a menu with mouse right click. At the bottom of the window is displayed information related to the specimen ID, scale, etc. Once a specimen is digitised, simply click on the arrow pointing toward the right in the menu bar to go to the next one. There is a possibility to use a “template mode” that copies a given configuration of landmarks across all pictures. This is convenient if the biological variation among pictures is subtle so that it causes minimum landmark adjustment across specimens (see ‘tpsDig2’ documentation for further information about this procedure). When saving the file, choose “save data,” name the file as a ‘.TPS’ file and click “save,” this brings you back to ‘tpsDig2’ and select “overwrite” to save the current version of the data.

^∗^Fourth, compile all .TPS files into a single one

The ‘.TPS’ files containing landmark coordinates from all imaging and digitising sessions need to be compiled into a single file. In ‘tpsUtil,’ select “Append files” to produce a compiled file and name it “FediaProjectCompiled.TPS.” Then, this file needs to be converted as an “.nts” file that will be later imported in MorphoJ for further analyses. In ‘tpsUtil,’ select “convert tps/nts coordinates file.” Select the ‘.TPS’ file that needs to be converted in “Input” (“FediaProjectCompiled.TPS”) and then select “Output” and give a name to the ‘.nts’ file that needs to be created (“FediaProjectCompiled.NTS”). Click “Create.” In “options,” tick “use scale factor” to convert the pixel coordinates into standard units of measurements via the scale factor that has been previously defined. In “No. dimensions,” select 2D landmarks. In “NTS and CSV labels” select “Image name.” There will be a preview at the bottom of the window that specifies the file to be created with the number of specimens and number of landmarks. Click “Create.” The newly created “.nts” file has the coordinates scaled and carries information about the labels defined for each picture. Thereafter, these labels can be read in MorphoJ or any related morphometric software to create classifiers or factors (e.g., population, weight, length, etc.) for further analyses.

Throughout this study, I will use MorphoJ for all analyses (Klingenberg, 2011). All procedures can also be carried out with the ‘tps’ suite of software, as well as other morphometrics-based R (R Core Team, 2018) packages such as ‘*geomorph*,’ ‘*shapes*,’ ‘*Morpho*,’ and ‘*Momocs*’ (Dryden and Dryden, 2012; Adams and Otárola-Castillo, 2013; Bonhomme et al., 2014; Schlager, 2017). MorphoJ is preferred to R packages or other software since it is probably the easiest standalone software to use. In addition, the graphical user interface is simple and clear and one can quickly run several analyses and generate fully customised graphs that can be exported as images or vectorised figures. Analyses of bilateral object symmetry are fully implemented, but not for more complex types of symmetry that are often found in flowers. Beyond bilateral symmetry, other solutions are needed and the R platform is suitable for this task. This is why I opt to operate data treatment for complex symmetry in R and use MorphoJ to analyse and visualise patterns of morphological variation.

## Dataset Preparation and Statistical Shape Analysis of Bilateral Symmetry (Zygomorphy), Two Perpendicular Axes of Symmetry (Bi- or Disymmetry), Rotational Symmetry Only, and Bilateral Symmetry Combined With Rotational Symmetry (Actinomorphy) in Flowers

### Case Study 1: Bilateral Object Symmetry in *F. graciliflora* (Symmetry Group C_1v_)

#### Data

I use wild-type flowers of *F. graciliflora* Fisch. and Meyer (Valerianaceae) as a case study (for details about rearing protocols, see Berger et al., 2017). *F. graciliflora* are fairly flat flowers with no visible veins, thus prior to any picture being taken they were immersed in a solution of 70% ethanol to reveal their venation system (**Figure 4C**). After the veins have been revealed, 30 flowers were photographed at the same magnification with a Lumenara camera (Model Infinity2-1C-ACS) mounted to a Zeiss Stemi-2000-C stereomicroscope. For each photograph, a configuration of 21 landmarks was acquired in two dimensions (**Figure 5A**) using tpsDig2 version 2.17. These landmarks are at the points of intersection between primary and secondary veins (Landmarks Type I) or at the maximum of curvature on the external outline of the flower (Landmarks Type II). Each picture was taken twice and each configuration of landmarks was digitised twice to test for measurement error due to imaging and digitising.

#### Bilateral Object Symmetry Analysis With MorphoJ

*Fedia graciliflora* flowers show a clear example of bilateral object symmetry also termed zygomorphy with the vertical axis of symmetry separating the dorsal petals and running through the ventral petal (described by landmarks 2, 7, 11, 13, and 19; **Figure 5A**).

Load the “FediaProjectCompiled.NTS” file in MorphoJ via “File – New Project,” name the project, then select “File-Create New Dataset.” This prompts a window to open in which the user selects the dimensionality of the data (select “2 dimensions”) and whether or not the data contains object symmetry (select “yes”), name the dataset (“FediaProjectCompiled.NTS”), and select the file type “NTSYSpc,” the name of the selected file appears in the field “File.” Click “Create Dataset.” The dataset is created and automatically contains all original configurations of landmarks with their reflected and appropriately relabelled copies. Create classifiers in the menu “Preliminaries” and choose “Extract new classifier from ID strings.” A new window appears in which a name for a new classifier can be entered. Create three classifiers for “Individual,” “Imaging,” and “Digitising” by entering a name for each of them. Then, the user needs to select the string of characters that corresponds to the length of a given classifier from the original identifier. For instance, the classifier for “Individual” that needs to be extracted from the identifier “Fedia01a1b1.jpg” should only comprise characters between the first and seventh digit which corresponds to “Fedia01.” This classifier for “Individual” can be extracted by entering “1” in the field for the first character and “7” in the field for the last character (reading from left to right). Follow the same procedure, by entering “8” and “9” to create the classifier “Imaging,” and “10” and “11” to define the classifier “Digitising.” Alternatively, one can also import already predefined classifiers created outside of MorphoJ as a separate file (see the MorphoJ on-line documentation for further details).

At this stage it is recommended to cheque for possible outliers before going into further analyses. Click “Preliminaries” and select “Find outliers.” This opens a tab in which the list of configurations is available and one can see the deviations of each configuration from the consensus. This is a quick way to identify aberrant configurations in the dataset. Outliers are often produced as a consequence of high measurement error or mislabelling of landmarks. After the search for outliers is completed and the data have been fixed, they can be superimposed by a GPA. To do so, click “Preliminaries” and select “New Procrustes Fit.” This prompts a window asking how the data should be presented. Select the default choice “align by principal axes” and click “Perform Procrustes fit.” A new tab appears representing the coordinates for the consensus (large blue dots) and the deviation around it that is symbolised by the superimposed configurations (small blue dots). At this point, MorphoJ has automatically separated the component of symmetric variation from the component of asymmetry and one can run analyses such as PCA on either component.

The components of symmetric variation and asymmetry can be exported by clicking on the dataset containing the superimposed Procrustes coordinates (“FediaProjectCompiled.NTS”), and by selecting “File” in the menu, then “Export Dataset.” The user is able to save the raw coordinates, the centroid sizes, and the superimposed coordinates for the symmetric component and asymmetry. This is useful if one wants to analyse these data with a different software.

##### PCA of bilateral object symmetry

To visualise the patterns of shape changes for each component, select “Preliminaries” and select “Generate Covariance Matrix,” the dataset which contains the superimposed Procrustes coordinates should be already selected, thereafter the user has the choice to select either the symmetric component or asymmetry. Select both components and click “Execute” without ticking the box “Pooled within-group covariances.” This generates the corresponding covariance matrices for each component and their patterns of variation are visualised via PCA by selecting “PCA” in the “Variation” menu. This creates a “Graphics” tab with three subtabs: “PC shape changes” that gives the patterns of shape variation for every PC (a right click on this tab gives access to several graphical options – Select “Change the Type of Graph” and choose “Warped Outline Drawing”), “Eigenvalues” (amount of variance explained by each PC) and “PC scores” (visualisation of individuals in the shape space). A “Results” tab is also produced that reports the results from the PCA (i.e., eigenvalues and PC coefficients).

##### Allometry

To test for allometry (i.e., influence of size on shape), a multivariate regression of the Procrustes coordinates onto centroid size is used by clicking on the original data set, then selecting “Regression” in the “Variation” menu (Klingenberg, 1996, 2016; Monteiro, 1999). In both left and right parts of the “Datasets” field, select the dataset “FediaProjectCompiled.NTS,” then in the left part of the field “Data matrices” select, for instance, the symmetric component “FediaProjectCompiled.NTS, symmetric component” and “FediaProjectCompiled.NTS, centroid size” in the right part of this field. In the field “Variables,” the user has the symmetric component in the left part and can choose between “Centroid Size” or “Log Centroid Size” in the right part. Tick the box “Perform permutation test” and select the number of rounds (e.g., 10,000). The option “Pooled regression within subgroups” is not needed here. Repeat the same procedure for the asymmetry if needed.

##### Measurement error

Select “File-Create New Dataset,” select “2 dimensions” and for object symmetry (select “no”), name the dataset (“FediaOriginal”), and select the file type “NTSYSpc,” the name of the selected file appears in the field “File.” Click “Create Dataset.” The dataset is created and contains all original configurations of landmarks only. Click on the newly generated dataset “FediaOriginal” then select “Variation” and go to “Procrustes ANOVA.” This will prompt a new window in which you can name the analysis and enter the main effects for your design that correspond to the classifiers previously created. Enter the classifier corresponding to “Individual,” Error 1 is the “Imaging” classifier and Error 2 is the “Digitising” classifier. Click “Execute.” This will create a results tab and covariance matrices for each effect in the project tree. Select all the covariance matrices (hold shift + left click). Go to the “Variation” menu and select “PCA” and run a PCA on each of the previously selected covariance matrices to visualise the corresponding patterns of shape changes for each effect.

#### Results From MorphoJ

##### PCA

There was a significant effect of size on shape for the symmetric (*P-*value < 0.0001), but not for the asymmetric component (*P-*value = 0.22). The regression of shape on centroid size accounted only for 7.61% and 1.16 % of total shape variation, respectively. Since allometry was very subtle for the component of symmetric variation, one can decide whether or not further analyses need to be carried on the residual component of the regression of shape on centroid size (Klingenberg, 1996, 2016; Monteiro, 1999).

The PCA on the symmetric component of shape variation reveals that adaxial petals show major displacements away from the axis of symmetry for PC1 (35.97% of variance), while PC2 exhibits reduction of adaxial petals and enlargement of abaxial ones (23.57% of variance). The PCA for the asymmetry suggests that all petals tend to bend in the overall same direction toward the right indicated by PC1 (60.45% of variance), adaxial petals do not exhibit much variation whereas abaxial petals show a preferential shift toward the right as opposed to the ventral petal as seen in PC2 (13.31% of variance) (**Figure 5B**).

Note that MorphoJ automatically separates the component of symmetric variation from the component of asymmetry, which means that when one runs a PCA on either component one cannot quantify how much variance is represented by these components with respect to the total amount of shape variation. Alternatively, a dataset already containing all original configurations and their reflected relabelled copies can be prepared outside of MorphoJ by using the R function ‘*C1v*’ (i.e., named after the Schoenflies notation of bilateral symmetry). A GPA of this imported dataset followed by a PCA will allow the user to determine how much variance is represented by the symmetric and asymmetric components with respect to the total amount of shape variation (see the Section “Bilateral Object Symmetry Analysis With R Using Function ‘*C1v*’).

##### Measurement error

The ANOVAs for centroid size and shape both reveal that the “Individual” main effect is highly significant (*P* < 0.0001), which means that the variation among flowers greatly exceeds the measurement error due to “Imaging” and “Digitising” (**Table 1**). The *F*-ratio for this effect indicates that the variation among flowers is almost 220 and 40 times larger than the measurement error due to imaging, respectively, for size and shape. The “Imaging” error term is significant (*P* < 0.001), which means that the imaging error is larger than the error due to digitising (nearly three times and twice larger according to the F-ratio for this effect, respectively, for size and shape). These results suggest that the biological variation at the population level largely exceeds all sources of measurement error due to imaging and digitising in my sample (**Table 1**).

**Table 1:** ANOVAs for measurement error for size and shape for case study 1: *F. graciliflora*.

Procrustes ANOVA					
Effect	SS	MS	*df*	*F*	*P*
Flower (variation among flowers)	0.81969197	0.0007438221	1102	40.68	<0.0001
Imaging (error due to taking images)	0.02084678	0.0000182866	1140	1.91	<0.0001
Digitising (error due to digitising landmarks)	0.02186079	0.0000095881	2280		
**Centroid size ANOVA**		
Flower (variation among flowers)	97.586684	3.365058	29	220.16	<0.0001
Imaging (error due to taking images)	0.458534	0.015284	30	2.80	0.0004
Digitising (error due to digitising landmarks)	0.328056	0.005468	60		

*SS, sum of squares; MS, mean square; Df, degrees of freedom; F, F-value; P, P-value.*

#### Bilateral Object Symmetry Analysis With R Using Function *‘C1v’*

As previously mentioned, MorphoJ does not give access to the full dataset made of all original configurations with their transformed and relabelled copies (see the Section “PCA”). Consequently, the user can carry out analyses on either the symmetric or asymmetric component of variation, but cannot quantify how much these components account for the total amount of shape variation. The solution is to import a prepared dataset that already contains the original configurations with the reflected relabelled copies. Here, I use the R function ‘*C1v*’ which applies bilateral object symmetry to a dataset containing all original configurations (**Figure 5A** and **Supplementary Material**). This function needs to be loaded in R prior to its use and must be applied to a two column matrix for 2D landmarks and a three column matrix for 3D landmarks containing the landmark coordinates only and that are arranged according to this sequence, respectively, x1, y1, x2, y2,…, xn, yn and x1, y1, z1, x2, y2, z2,…, xn, yn, zn. In addition, a “coFed.txt” file or object containing the relabelling information of landmarks for the reflected copy only needs to be created by the user so that it appropriately swaps the labels of the corresponding landmarks in the reflected copy (see “reflected relabelled copy” in **Figure 5A**, and “coFed.txt” file in **Supplementary Material**) and loaded in R. In this case, this ‘coFed’ element should be generated as a single column. ‘*C1v’* generates copies of all original configurations of landmarks and add them right after the matrix containing the original ones, then these copies are reflected with an appropriate relabelling of the landmarks indicated by the ‘coFed’ matrix producing a new doubled dataset with the first column containing the new identifiers as follows:

Ind001_a1b1_ori_Cn00
Ind001_a1b2_ori_Cn00
Ind001_a2b1_ori_Cn00
Ind001_a2b2_ori_Cn00
Ind001_a1b1_ref_Cn00
Ind001_a1b2_ref_Cn00
Ind001_a2b1_ref_Cn00
Ind001_a2b2_ref_Cn00

Reading from left to right, where ‘Ind001’ denotes the specimen, ‘a’ is the image and ‘1’ or ‘2’ is the repeat for a given imaging session, ‘b’ is the digitising and ‘1’ or ‘2’ refers to the repeat, ‘ori_Cn00’ denotes “reflection not applied (“ori”) and rotation not applied (“Cn00”) and therefore represents the original configuration only, ‘ref_Cn00’ denotes “reflected relabelled copy and no rotation applied” that is the reflected relabelled copy about the vertical left–right axis only.

The procedure to import and analyse this doubled dataset “RefRelabFedia.txt” in MorphoJ is the same as described in the Section “Bilateral Object Symmetry Analysis With MorphoJ” except that the user has to specify that there is no object symmetry in the data while importing them and the “text” file format must be selected. Thereafter, a GPA is performed on this dataset containing all original and reflected relabelled copies. A PCA on the total covariance matrix yields PCs either with symmetric or asymmetric shape changes. One can visually identify them by looking at their associated shape changes and subsequently group them according to specific categories of shape variation or inspecting specific patterns exhibited in the values and signs of the PC scores (see the Sections “Flowers With Object Symmetry Have a Special Mathematical Property” and “Analyses of Flowers With Object Symmetry Leads to Complex Structures in PCA Scatterplots” for details).

If one is only interested in carrying out analyses on the component of symmetric variation (i.e., variation among flowers) and wants to discard the asymmetry, one can calculate it by simply clicking on “Preliminaries” and selecting “Average Observations By,” then pick the appropriate dataset, select “Average by” and choose the classifiers corresponding to “Individual” or equivalent, with “all Data types” remaining selected. Click “execute.” This creates a new dataset with the same name as the original dataset with the extension “averaged” added to it. To visualise the patterns of morphological variation associated with this component, simply select this newly created dataset and go to “Preliminaries” then “Generate Covariance Matrix,” then go to “Variation” and choose “PCA.” See the Section “PCA of Bilateral Object Symmetry” for details regarding the output of this analysis. Measurement error due to imaging and digitising can be assessed via the procedure described in the Section “Measurement Error.”

#### Results From R Using Function *‘C1v’*

The PCA of the doubled dataset generated by the ‘*C1v*’ function yields 38 PCs. These PCs belong to two distinct categories of shape variation each containing 19 PCs. The number of PCs associated with either category of shape change is equally divided between these two classes for bilateral object symmetry due to geometric constraints imposed by the GPA (Mardia et al., 2000; Klingenberg et al., 2002). As an example, two PCs of each class of shape variation are shown (**Figure 5C**).

The first category of shape variation includes PCs that describe entirely asymmetric shape changes with respect to reflection about the left–right axis. For instance, PC1 exhibits shape changes with all petals bending toward either the left or right side, and PC9 shows opposite shifts of the left and right abaxial petals while the adaxial petals show much less variation in comparison. The 19 PCs of this category of shape variation represent 49.04% of the total variance.

The second category of shape variation includes PCs that describe fully symmetric shape changes with respect to reflection about the vertical left–right axis (i.e., zygomorphy). For instance, PC2 represents shape changes describing petal movements away from the vertical axis of symmetry while the ventral petal shows more subtle variation (in the positive direction), as opposed to PC5 that shows shape variation associated to a reduction of the overall size of the abaxial petals, by contrast to the adaxial petals, and an elongation of the ventral petal (in the positive direction). The 19 PCs of this category of shape variation represent 50.96% of the total variance. Recently, the non-model *F. graciliflora* flowers was used in a study combining geometric morphometrics and Virus Induced Gene Silencing (VIGS) to quantify the phenotypic effects of knocking down a single *CYC2* paralog, *FgCYC2A*, as well as the reporter gene, *FgANS* in symmetry of flowers (Berger et al., 2017). For a full step-by-step guide regarding all analyses carried in this case study, see **Supplementary Material**.

### Case Study 2: Decomposition of Corolla Shape Variation in *E. mediohispanicum* Flowers With Two Axes of Bilateral Symmetry (Bi-or Disymmetry), Equivalent to Reflection and Rotation of Order 2 (by 180°) (Symmetry Group C_2v_)

#### Data

The dataset was used in previous publications and for which 32 landmarks were digitised per flower, either at the points of intersection of primary and secondary veins with the petal margin or at the base of petals (for details, see Gómez et al., 2006; Savriama et al., 2012). Single digital photographs of 193 flowers from a unique wild population were taken.

#### Methods and Analyses

**FIGURE 6:**
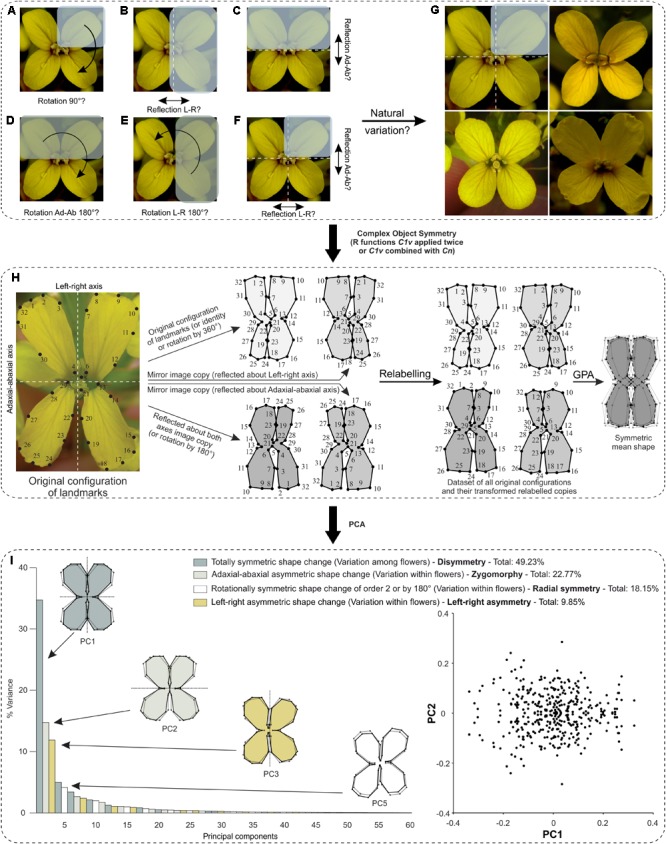
What is the biological repeated unit of interest in complex floral symmetry and how one should determine it? The case of *E. mediohispanicum* as an example. **(A–F)** Crucifers are symmetric with respect to two perpendicular axes of symmetry and several interpretations are possible concerning the identification of floral module(s) that generate the symmetry group of interest (indicated in parentheses). **(A)** Rotations by 90° (C_4_). **(B)** Reflection about the vertical left–right axis of either Adaxial-abaxial right or left petals (C_1_*_v_*). **(C)** Reflection about the horizontal adaxial–abaxial axis of either adaxial left–right petals or their abaxial equivalent (C_1_*_h_*). **(D)** Rotation by 180° about the centre of the flower of adaxial left–right petals or their abaxial (C_2_). **(E)** Rotation by 180° about the centre of the flower of adaxial–abaxial right petals or their left counterparts (C_2_). **(F)** Reflection combined with rotations by 180° (C_2_*_v_*). **(G)** The correct symmetry group was determined according to the well-known and in depth study of natural variation occurring in this flower which exhibits a wide range of different types of symmetry and corresponds to **(F)**. **(H,I)** Workflow for shape analysis of *E. mediohispanicum* flowers with two perpendicular axes of bilateral symmetry or bilateral symmetry combined with rotational symmetry of order 2 (by 180°) (case study 2). Credit: photograph courtesy of J. M. Gómez (Universidad de Granada, Spain). **(H)** First, an original configuration is considered, then all transformed relabelled copies in the symmetry group are produced either with function ‘*C1v*’ applied twice or after combining functions ‘*C1v*’ and ‘*Cn.*’ A GPA is applied to this dataset. The mean shape (consensus) is symmetric. **(I)** A PCA unambiguously separates the four components of shape variation and indicates how much their associated PCs account for the total variance. The first PC of each component is displayed. The outline wireframes of flowers show shape changes associated with each PC from the overall average shape (gray outline and open circles) for PC1 scores of –0.1 and +0.1, and for PC2 scores of –0.1 and +0.1 (solid black outline with coloured background and solid black circles). A scatterplot of the PC scores for PC1 and PC2 shows a distribution that is symmetric under reflection only. Each point in the plot corresponds to transformed copies of a given flower’s configuration of landmarks.

Because this flower as well as many other botanical systems possess complex symmetry (two perpendicular axes), this case study raises the issue on how one should analyse such structures. For instance, one can simply analyse bilateral object symmetry with respect to the centre of the flower (**Figure 6A**), or either axis of symmetry (**Figures 6B,C**), or only rotational object symmetry (**Figures 6D,E**), or both reflection and rotational object symmetry (**Figure 6F**). In this example, I already know that *E. mediohispanicum* flowers exhibit a wide range of different types of symmetry within a single wild population: bi- or disymmetry (symmetric with respect to any axis of symmetry), zygomorphy (symmetric regarding the left–right symmetry axis, but asymmetric about the adaxial–abaxial symmetry axis), actinomorphy (asymmetric to any axis of symmetry, but symmetric according to two successive reflections across both axes or equivalently to rotations by 180°), and left–right asymmetry (symmetric regarding the adaxial–abaxial symmetry axis, but asymmetric about the left–right symmetry axis) (**Figure 6G**). Therefore, object symmetry applied for the analysis of these flowers should take into account their complete symmetry group.

In this example, four transformed relabelled copies are needed per configuration: the original configuration (i.e., identity), a reflected copy about the vertical left–right axis, a reflected copy about the horizontal adaxial–abaxial axis, and a copy generated by two successive reflections about both axes (equivalent to a rotated copy by 180°) (**Figure 6H**).

First, the file “ErysimumRawData.TPS” is imported in R using the function ‘*readland.tps*’ and converted into a regular 2D data matrix with the function ‘*two.d.array*’ (both from the ‘*geomorph*’ package). Then, the “coEryLR.txt” file or object containing information about the relabelling of landmarks for reflection about the vertical left–right axis is imported. The reflected relabelled copies about the vertical left–right axis are generated first using the function ‘*C1v*,’ then the “coEryAdAb.txt” file or object containing information about the relabelling of landmarks for reflection about the horizontal adaxial–abaxial axis is imported. The reflected relabelled copies about the horizontal adaxial–abaxial axis as well as the transformed relabelled copies following two successive reflections (equivalent to rotation by 180°) are generated using the function ‘*C1v*’ a second time on the data previously generated by the first round. The newly produced dataset has the first column containing the new identifiers as follows:

001_ori_ori
001_ref_ori
001_ori_ref
001_ref_ref

Reading from left to right, where ‘001’ denotes the specimen, ‘ori_ori’ denotes “original and reflection not applied” and therefore represents the original configuration only, ‘ref_ori’ denotes “reflected relabelled copy” that is the reflected relabelled copy about the vertical left–right axis only, ‘ori_ref’ stands for “reflected relabelled copy” that is the reflected relabelled copy about the horizontal adaxial–abaxial axis only, and ‘ref_ref’ represents “reflected relabelled copy about both perpendicular axes or equivalently as rotation of order 2 (by 180°).”

Given that two successive reflections about these perpendicular axes are equivalent to rotation by 180°, an alternative solution also exists to generate the required transformed relabelled copies using the “coEry.txt” file. In this case, the reflected relabelled copies are generated first using the function ‘*C1v*,’ then these copies are further duplicated with an appropriate relabelling of landmarks according to each successive rotation using function ‘*Cn*’ (i.e., named after the symmetry group of rotational symmetry in Schoenflies notation) in a new dataset with the first column containing the new identifiers as follows:

001_ori_Cn00
001_ref_Cn00
001_ori_Cn01
001_ref_Cn01

Reading from left to right, where ‘001’ denotes the specimen, ‘ori_Cn00’ denotes “reflection not applied (“ori”) and rotation not applied (“Cn00”) and therefore represents the original configuration only, ‘ref_Cn00’ denotes “reflected relabelled copy and no rotation applied” that is the reflected relabelled copy about the vertical left–right axis only, ‘ori_Cn01’ stands for “reflection not applied, but rotation applied (by 180°)” that is the rotated relabelled copy only, ‘ref_Cn01’ represents “reflected relabelled copy and rotation applied (by 180°) that is the reflected relabelled copy about the horizontal left–right axis.”

In both solutions presented above, the procedure to import and analyse either “EryRefRefRelab.txt” or “EryRefRotRelab.txt,” which already contains all original and their transformed relabelled copies, in MorphoJ is the same as described in the Section “Bilateral Object Symmetry Analysis With R Using Function ‘*C1v*’.” A single GPA on this dataset is carried in MorphoJ. A PCA separates out the different components of symmetric and asymmetric variations in the data. These different components can be isolated and grouped together into the same categories that account for the same shape changes [see the Section “Flowers With Symmetric, but Physically Connected Petals (Object Symmetry)”].

#### Results

Here is a summary of the results that were already published in Savriama et al. (2012). The PCA of the full dataset yields 60 PCs. These PCs can be unambiguously allocated to four categories of shape variation, with each of them containing 15 PCs (the number of PCs associated with either class of shape change is equally divided among them in this particular case). As an example, the first two PCs of each class of shape variation are shown in **Figure 6I**.

The first category of shape variation includes PCs that describe entirely symmetric shape changes with respect to reflection about the left–right axis, the horizontal adaxial–abaxial axis, and two successive reflections about both axes (equivalent to rotations by 180°). For instance, PC1 exhibits shape changes describing shifts between generally rectangular and square flowers. The 15 PCs of this category of shape variation represent 49.23% of the total variance.

The second category of shape variation includes PCs that describe asymmetric shape changes regarding reflection with respect to the horizontal adaxial–abaxial axis, but symmetric according to reflection about the left–right axis (i.e., zygomorphy). For instance, PC2 represents shape changes describing petal movements away from the vertical axis of symmetry for the adaxial compartment, while the opposite is happening for the abaxial unit (in the positive direction). The 15 PCs of this category of shape variation represent 22.77% of the total variance.

The third class of morphological variation is related to shape changes that are asymmetric regarding the left–right axis, but symmetric relative to the adaxial–abaxial axis. For example, PC3 exhibits shape changes with all petals bending toward either the left or right side. The 15 PCs of this category of morphological variation account for 18.15% of the total variance.

The fourth and last class of morphological variation contains PCs associated with shape changes that are asymmetric regarding either axis of bilateral symmetry, but symmetric relative to rotations by 180° about the centre located at the intersection of these axes. For instance, PC5 corresponds to shape changes that show alternate and opposite expansions and simultaneous reductions of petals facing each other in their diagonal counterparts. The 15 PCs of this ensemble of morphological changes together sum up to 9.85% of the total variance.

A scatterplot of the scores for PC1 vs. PC2 shows a distribution that has bilateral symmetry with respect to the horizontal axis. This is because the PC1 represents completely symmetric shape changes while PC2 is associated with symmetric shape changes with respect to the left–right axis only (see the Sections “Flowers With Object Symmetry Have a Special Mathematical Property” and “Analyses of Flowers With Object Symmetry Leads to Complex Structures in PCA Scatterplots”). This approach has been used to test the hypothesis suggested by developmental genetic studies of symmetry in plants that adaxial–abaxial asymmetry in crucifers is more easily generated in floral development than left–right asymmetry and should be more abundant (Savriama et al., 2012) and examine in deeper detail the drastic morphological changes associated with shift from outcrossing to selfing (Carleial et al., 2017). For a full step-by-step guide regarding all analyses carried in this case study, see **Supplementary Material**.

### Case Study 3: Decomposition of Corolla Shape Variation in *Vinca minor* (Apocynaceae) Flowers With Rotational Symmetry of Order 5 (Symmetry Group C_5_)

#### Data

**FIGURE 7:**
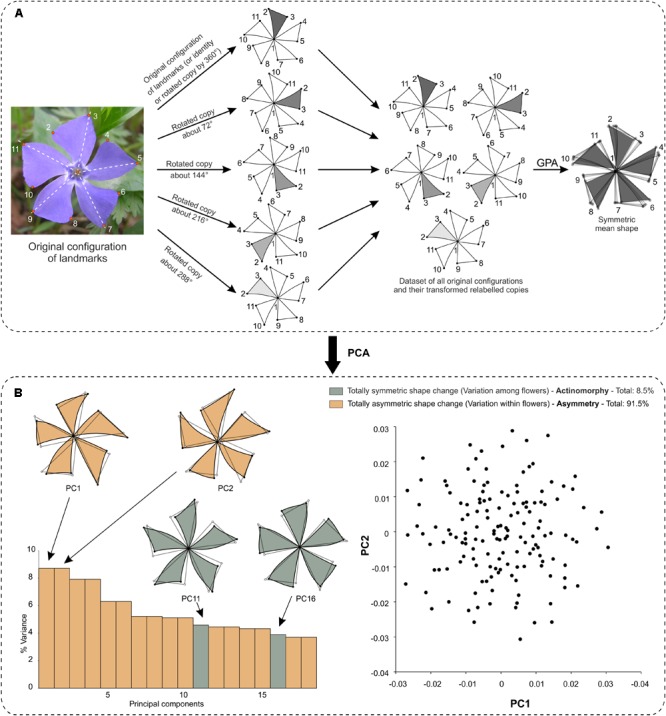
Workflow for shape analysis of the pinwheel *V. minor* with rotational symmetry of order 5 (by 72°) (case study 3). **(A)** First, an original configuration is considered, then all transformed relabelled copies in the symmetry group are produced with function ‘*Cn.*’ A GPA is applied to this dataset. The mean shape (consensus) is symmetric. **(B)** A PCA unambiguously separates the two components of shape variation and indicates how much their associated PCs account for the total variance. The first two PCs of each component was displayed. The shape changes for pairs of PCs with equal eigenvalues are totally asymmetric. The outline soft wireframes of flowers are as in **Figure 6**. A scatterplot of the PC scores for the first two PCs shows a distribution that is symmetric under rotation of order 5. Each point in the plot corresponds to transformed copies of a given flower’s configuration of landmarks. Credit: photograph by Beentree, distributed under a Creative Commons Attribution-Share Alike 3.0 Unported licence.

I analysed a simulated dataset of 11 landmarks in 2D for 30 individual pinwheel flowers with a small amount of independent and isotropic variation around each landmark. The original configuration of landmarks from which the data were simulated was acquired on a single digital photograph (**Figure 7A**).

#### Methods and Analyses

In this example, five transformed relabelled copies are needed per configuration: the original configuration (i.e., identity), a rotated copy by 72° (1^∗^2pi/5), a rotated copy by 144°, a rotated copy by 216°, and a rotated copy by 288° (**Figure 7A**).

First, the file “VincaRawSimul30.txt” is imported in R using the function ‘*read.table.*’ Then, the “coVin.txt” file or object containing information about the relabelling of landmarks for rotation is imported. This “coVin” element needs to be created by the user so that it appropriately swaps the labels of the corresponding landmarks in the successive copies according to each rotation (see **Figure 7A**, and “coVin.txt” file in **Supplementary Material**). The columns in ‘*coVin*’ should contain the relabelling information regarding the successive rotations. The rotated relabelled copies are generated using function ‘*Cn*’ in a new dataset with the first column containing the new identifiers as follows:

ind1_Cn00
ind1_Cn01
ind1_Cn02
ind1_Cn03
ind1_Cn04

Reading from left to right, where ‘ind1’ denotes the specimen, ‘Cn00’ denotes “rotation not applied or rotation by 360° applied” and therefore represents the original configuration only, ‘Cn01’ stands for “rotation applied (by 72°)” that is the rotated relabelled copy only, ‘Cn02’ stands for “rotation applied (by 144°)” that is the rotated relabelled copy only, ‘Cn03’ stands for “rotation applied (by 216°)” that is the rotated relabelled copy only, ‘Cn04’ stands for “rotation applied (by 288°)” that is the rotated relabelled copy only.

The procedure to import and analyse this dataset, which already contains all original and their transformed relabelled copies, in MorphoJ is the same as described in the Section “Bilateral Object Symmetry Analysis With R Using Function ‘*C1v*’.” A single GPA on this dataset is carried in MorphoJ.

#### Results

A PCA on the covariance matrix of the Procrustes tangent coordinates obtained from the “VincaRawSimul30.txt” dataset yields 18 PCs. Of these PCs, many occur as pairs and each PC of a pair, taken separately, represent shape changes that are totally asymmetric (see the Section “Flowers With Symmetric, but Physically Connected Petals (Object Symmetry)” for details). There are eight pairs of them that represent 91.5% of variance. The other two remaining PCs show shape changes that are completely symmetric with respect to rotation of order 5 (by 72°) accounting for 8.5% of total variance. A scatterplot of the PC scores for the pairs of PCs with equal eigenvalues shows a distribution of the scores that has rotational symmetry of order 5 (**Figure 7B**). This is because PC1 and PC2 both represent completely asymmetric shape changes with respect to rotational symmetry of order 5 (or by 72°) (see the Sections “Flowers With Object Symmetry Have a Special Mathematical Property” and “Analyses of Flowers With Object Symmetry Leads to Complex Structures in PCA Scatterplots”). For a full step-by-step guide regarding all analyses carried in this case study, see **Supplementary Material**.

### Case Study 4: Decomposition of Corolla Shape Variation in *Trillium undulatum* (Melanthiaceae) Flowers With Reflection and Rotational Symmetry of Order 3 (Symmetry Group C_3v_)

#### Data

**FIGURE 8:**
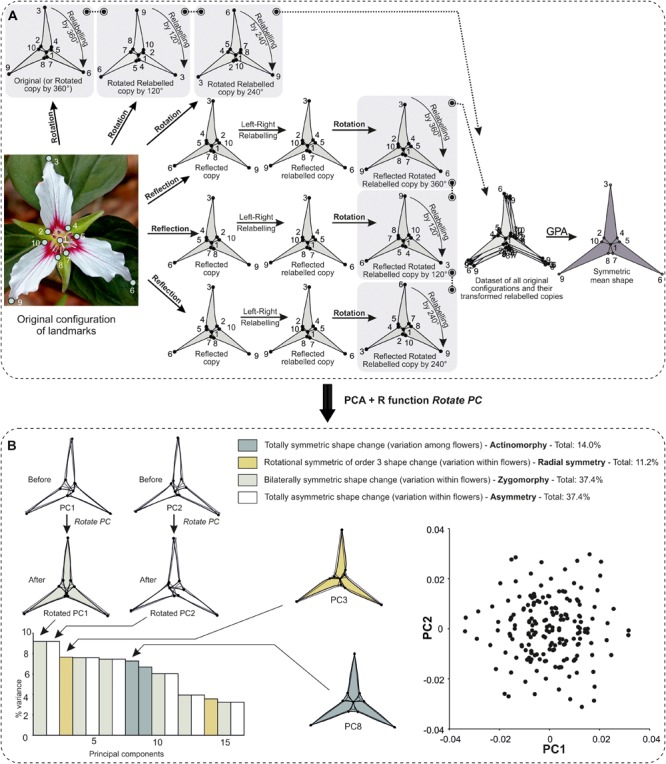
Workflow for shape analysis of *T. undulatum* exhibiting bilateral symmetry combined with rotational symmetry of order 3 (by 120°) (case study 4). **(A)** First, an original configuration is considered, then all transformed relabelled copies in the symmetry group are produced with functions ‘*C1v*’ and ‘*Cn.*’ A GPA is applied to this dataset. The mean shape (consensus) is symmetric. **(B)** A PCA unambiguously separates the four categories of shape variation and indicates how much their associated PCs account for the total variance. The first PC of each category is displayed. The shape changes for pairs of PCs with equal eigenvalues that first appear as totally asymmetric are further revealed by appropriately rotating them using function ‘*RotatePC.*’ The outline soft wireframes of flowers are as in **Figure 6**. A scatterplot of the PC scores for the first two PCs shows a distribution that is symmetric under reflection combined with rotation of order 3. Each point in the plot corresponds to transformed copies of a given flower’s configuration of landmarks. Credit: photograph by Nicholas A. Tonelli, distributed under a cc-by-2.0 licence.

I analysed a dataset that contained simulated 2D configurations of 10 landmarks for 30 individual flowers with a small amount of independent and isotropic variation around each landmark. The original configuration of landmarks from which the data were simulated was acquired on a single digital photograph (**Figure 8A**).

#### Methods and Analyses

In this example, six transformed relabelled copies are needed: the original configuration (i.e., identity), rotated copy by 120°, rotated copy by 240°, reflected copy about the vertical left–right axis, reflected copy about the vertical left–right axis combined with a rotation by 120°, and reflected copy about the vertical left–right axis combined with a rotation by 240° (**Figure 8A**).

First, the file “TrilliumRawSimul30.txt” is imported in R using the function ‘*read.table.*’ Then, the “coTri.txt” file or object containing information about the relabelling of landmarks for reflection and rotation is imported. This “coTri” element needs to be created by the user so that it appropriately swaps the labels of the corresponding landmarks in the copies according to reflection, rotations and combination of both (see **Figure 8A** and “coTri.txt” file in **Supplementary Material**). The first column in ‘co.Tri’ should contain the relabelling information about the reflection and the rest of the columns include the relabelling regarding the successive rotations. The reflected relabelled copies are generated first using the function ‘*C1v*,’ then these copies as well as the original configurations are further duplicated and transformed relabelled according to each successive rotation using function ‘*Cn.*’ The newly produced dataset has the first column containing the new identifiers as follows:

ind1_ori_Cn00
ind1_ref_Cn00
ind1_ori_Cn01
ind1_ref_Cn01
ind1_ori_Cn02
ind1_ref_Cn02

Reading from left to right, where ‘001’ denotes the specimen, ‘ori_Cn00’ denotes “reflection not applied (“ori”) and rotation not applied (“Cn00”) and therefore represents the original configuration only, ‘ref_Cn00’ denotes “reflected relabelled copy and no rotation applied” that is the reflected relabelled copy about the vertical left–right axis only, ‘ori_Cn01’ stands for “reflection not applied, but rotation applied (by 120°)” that is the rotated relabelled copy only, ‘ref_Cn01’ represents “reflected relabelled copy and rotation applied (by 120°), ‘ori_Cn02’ stands for “reflection not applied, but rotation applied (by 240°)” that is the rotated relabelled copy only, ‘ref_Cn02’ represents “reflected relabelled copy and rotation applied (by 240°).

The procedure to import and analyse this dataset, which already contains all original and their transformed relabelled copies, in MorphoJ is the same as described in the Section “Bilateral Object Symmetry Analysis With R Using Function ‘*C1v*’.” A single GPA on this dataset is carried in MorphoJ.

#### Results

A PCA on the covariance matrix of the Procrustes tangent coordinates obtained from the “RefRotRelabTri.txt” dataset yields 16 PCs. Of these PCs, many occur as pairs with equal eigenvalues (**Figure 8B** and see the Sections “Flowers With Object Symmetry Have a Special Mathematical Property” and “Analyses of Flowers With Object Symmetry Leads to Complex Structures in PCA Scatterplots” for details). Two PCs are associated with shape changes that are completely symmetric (bilateral symmetry combined with rotational symmetry of order 3 or by 120°). Two PCs represent shape changes that are symmetric with respect to rotation of order 3 (or by 120°) only. There are 12 pairs of PCs and each PC of a pair, taken separately, appear asymmetric as it is expected for flowers with rotational symmetry of order higher than 2. A solution to unveil the shape changes represented by each PC belonging to a pair with equal eigenvalues is to appropriately rotate them (see the Section “Flowers With Object Symmetry Have a Special Mathematical Property”). This is accomplished via the R function ‘*RotatePC.*’ The newly rotated PCs “RotatedEvec.txt” can be imported for visualisation in MorphoJ and are linked with the original dataset (**Figure 8B**). After being appropriately rotated, each pair of PCs with equal eigenvalues now has one of the PC that shows shape changes that are bilaterally symmetric (37.4% of total variance), while the other one exhibits shape changes that are totally asymmetric (37.4% of total variance). The PCs with single eigenvalues either represent shape changes with reflection and rotational symmetry of order 3 (14.0% of total variance) or rotational symmetry of order 3 only (11.2% of total variance). A scatterplot of the scores for the pairs of PCs with equal eigenvalues shows a distribution that is a combination of bilateral symmetry and rotational symmetry of order 3 (or by 120°). This is because PC1 is associated with shape changes that are bilaterally symmetric and PC2 represent completely asymmetric shape changes (see the Sections “Flowers With Object Symmetry Have a Special Mathematical Property” and “Analyses of Flowers With Object Symmetry Leads to Complex Structures in PCA Scatterplots”). For a full step-by-step guide regarding all analyses carried in this case study, see **Supplementary Material**.

## Conclusion

The protocols described above provide step-by-step instructions for the analysis of corolla shape and of its symmetries. It unambiguously allows detecting, partitioning and quantifying the different symmetric and asymmetric components of morphological variation exhibited in populations of flowers, so far mostly classified with the naked eye and typological approaches. This set of approaches is particularly relevant for researchers interested in flower shape variation, evolution, and developmental biology. It is also of interest for biologists working on (i) serial homology in leaves since plants often have repeated organs that are arranged according to symmetric patterns, (ii) chirality in floral organs (rotational twisted growth of structures in a preferred direction as well as its opposite), (iii) enantiostyly in flowers in which the style points alternatively in a preferred direction consequently creating flowers that are mirror images of each other.

Other methods further decompose the asymmetric variation into fluctuating asymmetry (FA) and directional asymmetry (DA). FA refers to small random differences among repeated parts of individuals) that is often used as an estimate of developmental instability (i.e., the inability of a genome to buffer or correct for developmental perturbations) or as a tool to infer patterns of developmental modularity and phenotypic integration (Klingenberg, 2003). DA stands for the mean asymmetry or population asymmetry when on average one part or a set of parts is/are consistently more developed than the other(s). A two-way mixed model Procrustes ANOVA has been developed in this context (Leamy, 1984; Palmer and Strobeck, 1986; Klingenberg and McIntyre, 1998; Klingenberg et al., 2002). This framework has been generalised for the study of any type of symmetric structure (Savriama and Klingenberg, 2011; Savriama et al., 2012; Savriama and Gerber, 2018), but still represents a considerable challenge to implement due to the large diversity and increasing complexity of designs that can be generated according to the type of symmetry under study. Also, due to the particular structuration of the shape tangent space for flowers with object symmetry, Procrustes ANOVA designs pose conceptual and statistical subtleties, as opposed to the same approaches for flowers with matching symmetry (Savriama and Klingenberg, 2011; Savriama and Gerber, 2018). In addition, while the concept of ‘target phenotype’ (i.e., the phenotype characterised by the genetic makeup of the organism and the environmental conditions during its development) (Nijhout and Davidowitz, 2003) is obvious for bilateral symmetry it is not directly generalised for more complex and ambiguous types of symmetry. Therefore, this particular approach is far beyond the scope of this guide, while the method presented here that uses PCA to decompose the variation according to separate components of symmetry/asymmetry is of more general appeal to a wider audience and has been already applied in different botanical studies (Potapova and Hamilton, 2007; Poulíčková et al., 2010; Savriama et al., 2010, 2012; Neustupa, 2013; Carleial et al., 2017).

Studies combining geometric morphometrics with developmental genetics open a wide range of new potential applications to further understand the origins of morphological variation in flowers and the role of symmetry/asymmetry in the evolutionary success of angiosperms. For instance, a newly published study coupled geometric morphometrics and VIGS to quantify the phenotypic effects of knocking down a single *CYCLOIDEA* (CYC2) paralog, *FgCYC2A*, as well as the reporter gene, *ANTHOCYANIDIN SYNTHASE* (*FgANS*) in symmetry of flowers (Berger et al., 2017). This protocol will therefore become an essential protocol within the toolkit for botanists.

## Dedication

For Amy Buckner Reichbind, in memoriam.

## Author Contributions

The author confirms being the sole contributor of this work and approved it for publication.

## Conflict of Interest Statement

The authordeclares that the research was conducted in the absence of any commercial or financial relationships that could be construed as a potential conflict of interest. The reviewer EMC and handling Editor declared their shared affiliation.

## References

[B1] Abdi H. Williams L. J. (2010). Principal component analysis. *Wiley Interdiscip. Rev.* 2 433–459. 10.1002/wics.101

[B2] Adams D. C. Otárola-Castillo E. (2013). geomorph: an R package for the collection and analysis of geometric morphometric shape data. *Methods Ecol. Evol.* 4 393–399. 10.1111/2041-210X.12035

[B3] Almeida J. Rocheta M. Galego L. (1997). Genetic control of flower shape in *Antirrhinum majus*. *Development* 124 1387–1392. 9118809 10.1242/dev.124.7.1387

[B4] Armstrong M. A. (2013). *Groups and Symmetry.* Berlin: Springer Science & Business Media.

[B5] Arnqvist G. Martensson T. (1998). Measurement error in geometric morphometrics: empirical strategies to assess and reduce its impact on measures of shape. *Acta Zool. Acad. Sci. Hung.* 44 73–96.

[B6] Azani N. Babineau M. Bailey C. D. Banks H. Barbosa A. R. Pinto R. B. (2017). A new subfamily classification of the Leguminosae based on a taxonomically comprehensive phylogeny The Legume Phylogeny Working Group (LPWG). *Taxon* 66 44–77. 10.12705/661.3

[B7] Backlund A. Donoghue M. (1996). “Morphology and phylogeny of the order Dipsacales,” in *Phylogeny of the Dipsacales* ed. Backlund A. A. (Uppsala: Uppsala University) 243:06.

[B8] Bailey R. C. Byrnes J. (1990). A new, old method for assessing measurement error in both univariate and multivariate morphometric studies. *Systemat. Zool.* 39 124–130. 10.2307/2992450

[B9] Bateman R. M. Rudall P. J. (2006). Evolutionary and morphometric implications of morphological variation among flowers within an inflorescence: a case-study using European orchids. *Ann. Bot.* 98 975–993. 10.1093/aob/mcl191 17018569 PMC2803595

[B10] Berger B. A. Ricigliano V. A. Savriama Y. Lim A. Thompson V. Howarth D. G. (2017). Geometric morphometrics reveals shifts in flower shape symmetry and size following gene knockdown of CYCLOIDEA and ANTHOCYANIDIN SYNTHASE. *BMC Plant Biol.* 17:205. 10.1186/s12870-017-1152-x 29149840 PMC5693587

[B11] Berger B. A. Thompson V. Lim A. Ricigliano V. Howarth D. G. (2016). Elaboration of bilateral symmetry across *Knautia macedonica* capitula related to changes in ventral petal expression of CYCLOIDEA-like genes. *EvoDevo* 7:8. 10.1186/s13227-016-0045-7 27042288 PMC4818532

[B12] Bonhomme V. Picq S. Gaucherel C. Claude J. (2014). Momocs: outline analysis using R. *J. Statist. Softw.* 56 1–24. 10.18637/jss.v056.i13

[B13] Bookstein F. L. (1997). Landmark methods for forms without landmarks: morphometrics of group differences in outline shape. *Med. Image Anal.* 1 225–243. 10.1016/S1361-8415(97)85012-8 9873908

[B14] Burzlaff H. Zimmermann H. (2006). “Point-group symbols,” in *International Tables for Crystallography Volume A: Space-group symmetry* ed. Carolyn P. (Berlin: Springer) 742–749.

[B15] Busch A. Zachgo S. (2007). Control of corolla monosymmetry in the Brassicaceae *Iberis amara*. *Proc. Natl. Acad. Sci. U.S.A.* 104 16714–16719. 10.1073/pnas.0705338104 17940055 PMC2034219

[B16] Carleial S. Van Kleunen M. Stift M. (2017). Small reductions in corolla size and pollen: ovule ratio, but no changes in flower shape in selfing populations of the North American *Arabidopsis lyrata*. *Oecologia* 183 401–413. 10.1007/s00442-016-3773-4 27866292

[B17] Carlson S. E. Howarth D. G. Donoghue M. J. (2011). Diversification of *CYCLOIDEA*-like genes in Dipsacaceae (Dipsacales): implications for the evolution of capitulum inflorescences. *BMC Evol. Biol.* 11:325. 10.1186/1471-2148-11-325 22054400 PMC3224765

[B18] Citerne H. Jabbour F. Nadot S. Damerval C. (2010). The evolution of floral symmetry. *Adv. Bot. Res.* 54 85–137. 10.1016/S0065-2296(10)54003-5

[B19] Conway J. H. Burgiel H. Goodman-Strauss C. (2016). *The Symmetries of Things.* Boca Raton, FL: CRC Press.

[B20] Dryden I. Dryden M. I. (2012). *Shapes package.* Vienna: R Foundation for Statistical Computing.

[B21] Dryden I. L. Mardia K. V. (1998). *Statistical Shape Analysis.* Chichester: John Wiley.

[B22] Endress P. K. (2001). Evolution of floral symmetry. *Curr. Opin. Plant Biol.* 4 86–91. 10.1016/S1369-5266(00)00140-0 11163173

[B23] Flurry R. L. (1980). *Symmetry Groups: Theory and Chemical Applications.* Upper Saddle River, NJ: Prentice Hall.

[B24] Frey F. M. Bukoski M. (2014). Floral symmetry is associated with flower size and pollen production but not insect visitation rates in *Geranium robertianum* (Geraniaceae). *Plant Species Biol.* 29 272–280. 10.1111/1442-1984.12021

[B25] Frey F. M. Robertson A. Bukoski M. (2007). A method for quantifying rotational symmetry. *New Phytol.* 175 785–791. 10.1111/j.1469-8137.2007.02146.x 17688593

[B26] Fruciano C. (2016). Measurement error in geometric morphometrics. *Dev. Genes Evol.* 226 139–158. 10.1007/s00427-016-0537-4 27038025

[B27] Gómez J. M. Perfectti F. (2010). Evolution of complex traits: the case of *Erysimum* corolla shape. *Int. J. Plant Sci.* 171 987–998. 10.1086/656475

[B28] Gómez J. M. Perfectti F. Camacho J. P. M. (2006). Natural selection on *Erysimum mediohispanicum* flower shape: insights into the evolution of zygomorphy. *Am. Nat.* 168 531–545. 10.1086/507048 17004224

[B29] Goodall C. (1991). Procrustes methods in the statistical analysis of shape. *J. R. Statist. Soc. Ser. B* 53 285–339.

[B30] Gunz P. Mitteroecker P. (2013). Semilandmarks: a method for quantifying curves and surfaces. *Hystrix Ital. J. Mammal.* 24 103–109.

[B31] Gunz P. Mitteroecker P. Bookstein F. L. (2005). “Semilandmarks in three dimensions,” in *Modern Morphometrics in Physical Anthropology* ed. Dennis E. (Berlin: Springer) 73–98. 10.1007/0-387-27614-9_3

[B32] Hallgrimsson B. Percival C. J. Green R. Young N. M. Mio W. Marcucio R. (2015). Morphometrics, 3D imaging, and craniofacial development. *Curr. Top. Dev. Biol.* 115 561–597. 10.1016/bs.ctdb.2015.09.003 26589938 PMC5299999

[B33] Hernández-Ramírez A. M. Aké-Castillo J. A. (2014). A Geometric morphometrics study of stigma-anther polymorphism in the tropical Distylous *Palicourea padifolia* (Rubiaceae). *Am. J. Plant Sci.* 5 1449–1458. 10.4236/ajps.2014.510160

[B34] Herrera C. M. (1993). Selection on floral morphology and environmental determinants of fecundity in a hawk moth-pollinated violet. *Ecol. Monograp.* 63 251–275. 10.2307/2937101

[B35] Jolliffe I. T. (2002). *Principal Component Analysis* 2nd Edn. Berlin: Springer-Verlag.

[B36] Kent J. T. Mardia K. V. (2001). Shape, Procrustes tangent projections and bilateral symmetry. *Biometrika* 88 469–485. 10.1093/biomet/88.2.469

[B37] Klingenberg C. P. (1996). “Multivariate allometry,” in *Advances in Morphometrics* eds Marcus L. F. Corti M. Loy A. Naylor G. J. P. Slice D. E. (Berlin: Springer) 23–49. 10.1007/978-1-4757-9083-2_3

[B38] Klingenberg C. P. (2003). “Developmental instability as a research tool: using patterns of fluctuating asymmetry to infer the developmental origins of morphological integration,” in *Developmental Stability: Causes and Consequences* ed. Polak M. (Oxford: Oxford University Press) 427–442.

[B39] Klingenberg C. P. (2011). MorphoJ: an integrated software package for geometric morphometrics. *Mol. Ecol. Resour.* 11 353–357. 10.1111/j.1755-0998.2010.02924.x 21429143

[B40] Klingenberg C. P. (2016). Size, shape, and form: concepts of allometry in geometric morphometrics. *Dev. Genes Evol.* 226 113–137. 10.1007/s00427-016-0539-2 27038023 PMC4896994

[B41] Klingenberg C. P. Barluenga M. Meyer A. (2002). Shape analysis of symmetric structures: quantifying variation among individuals and asymmetry. *Evolution* 56 1909–1920. 10.1111/j.0014-3820.2002.tb00117.x 12449478

[B42] Klingenberg C. P. McIntyre G. S. (1998). Geometric morphometrics of developmental instability: analyzing patterns of fluctuating asymmetry with Procrustes methods. *Evolution* 52 1363–1375. 10.1111/j.1558-5646.1998.tb02018.x 28565401

[B43] Kolamunnage R. Kent J. T. (2003). “Principal component analysis for shape variation about an underlying symmetric shape,” in *Proceedings of the Stochastic Geometry, Biological Structure and Images* eds Aykroyd R.G. Langdon K. V. Mardia M. J. (Leeds: Leeds University Press) 137–139.

[B44] Kolamunnage R. Kent J. T. (2005). Decomposing departures from bilateral symmetry. *Quant. Biol. Shape Analysis Wavelets* 75–78.

[B45] Leamy L. (1984). Morphometric studies in inbred and hybrid house mice. V. Directional and fluctuating asymmetry. *Am. Natural.* 123 579–593. 10.1086/284225

[B46] Lindley J. (1836). *Edwards’s Botanical Register* Vol. 22. London: James Ridgway and Sons.

[B47] Luo D. Carpenter R. Copsey L. Vincent C. Clark J. Coen E. (1999). Control of organ asymmetry in flowers of Antirrhinum. *Cell* 99 367–376. 10.1016/S0092-8674(00)81523-8 10571179

[B48] Mardia K. V. Bookstein F. L. Moreton I. J. (2000). Statistical assessment of bilateral symmetry of shapes. *Biometrika* 2 285–300. 10.1016/j.joen.2017.01.012 28377148

[B49] Martin G. E. (2012). *Transformation Geometry: An Introduction to Symmetry.* Berlin: Springer Science & Business Media.

[B50] Møller A. P. Swaddle J. P. (1997). *Asymmetry, Developmental Stability and Evolution.* Oxford: Oxford University Press.

[B51] Monteiro L. R. (1999). Multivariate regression models and geometric morphometrics: the search for causal factors in the analysis of shape. *Systemat. Biol.* 48 192–199. 10.1080/106351599260526 12078640

[B52] Nattero J. Cocucci A. Medel R. (2010). Pollinator-mediated selection in a specialized pollination system: matches and mismatches across populations. *J. Evol. Biol.* 23 1957–1968. 10.1111/j.1420-9101.2010.02060.x 20695967

[B53] Neustupa J. (2013). Patterns of symmetric and asymmetric morphological variation in unicellular green microalgae of the genus *Micrasterias* (Desmidiales, Viridiplantae). *Fottea* 13 53–63. 10.5507/fot.2013.005

[B54] Nijhout H. F. Davidowitz G. (2003). “Developmental perspectives on phenotypic plasticity, canalization, and fluctuating asymmetry,” in *Developmental Instability: Causes and Consequences* ed. Polak M. (Cambridge, MA: MIT Press) 3–13.

[B55] Palmer A. R. Strobeck C. (1986). Fluctuating asymmetry: measurement, analysis, patterns. *Annu. Rev. Ecol. Systemat.* 17 391–421. 10.1146/annurev.es.17.110186.002135

[B56] Polak M. (2003). *Developmental Instability: Causes and Consequences.* Oxford: Oxford University Press on Demand.

[B57] Potapova M. Hamilton P. B. (2007). Morphological and ecological variation within the *Achnanthidium minutissimum* (Bacillariophyceae) species complex 1. *J. Phycol.* 43 561–575. 10.1111/j.1529-8817.2007.00332.x

[B58] Poulíčková A. Veselá J. Neustupa J. Škaloud P. (2010). Pseudocryptic diversity versus cosmopolitanism in diatoms: a case study on *Navicula cryptocephala* Kütz.(Bacillariophyceae) and morphologically similar taxa. *Protist* 161 353–369. 10.1016/j.protis.2009.12.003 20097131

[B59] Radović S. Urošević A. Hočevar K. Vuleta A. Jovanović S. M. Tucić B. (2017). Geometric morphometrics of functionally distinct floral organs in Iris pumila: analyzing patterns of symmetric and asymmetric shape variations. *Arch. Biol. Sci.* 69 223–231. 10.2298/ABS160912086R

[B60] Raven P. H. (1977). Onagraceae. *Flora Malesiana Ser. 1 Spermat.* 8 99–107.

[B61] R Core Team (2018). *R: A Language and Environment for Statistical Computing.* Vienna: R Foundation for Statistical Computing Available at: https://www.R-project.org/

[B62] Rix M. (2010). Tropaeolaceae: 686. *TROPAEOLUM SPECIOSUM*. *Curtis’s Bot. Mag.* 27 290–295. 10.1111/j.1467-8748.2010.01705.x

[B63] Rodríguez I. Gumbert A. De Ibarra N. H. Kunze J. Giurfa M. (2004). Symmetry is in the eye of the ‘beeholder’: innate preference for bilateral symmetry in flower-naïve bumblebees. *Naturwissenschaften* 91 374–377. 10.1007/s00114-004-0537-5 15278213

[B64] Rohlf F. J. (2015). The tps series of software. *Hystrix* 26 9–12.

[B65] Rohlf F. J. Slice D. (1990). Extensions of the Procrustes method for the optimal superimposition of landmarks. *Systemat. Biol.* 39 40–59.

[B66] Rosen J. (1975). *Symmetry Discovered: Concepts and Applications in Nature and Science.* North Chelmsford, MA: Courier Corporation.

[B67] Sargent R. D. (2004). Floral symmetry affects speciation rates in angiosperms. *Proc. R. Soc. Lond. B Biol. Sci.* 271 603–608. 10.1098/rspb.2003.2644 15156918 PMC1691633

[B68] Sauquet H. Von Balthazar M. Magallón S. Doyle J. A. Endress P. K. Bailes E. J. (2017). The ancestral flower of angiosperms and its early diversification. *Nat. Commun.* 8:16047. 10.1038/ncomms16047 28763051 PMC5543309

[B69] Savriama Y. Gerber S. (2018). Geometric morphometrics of nested symmetries: hierarchical inter-and intra-individual variation in biological shapes. *bioRxiv* [Pre print] 10.1101/306712 PMC6303334 30575747

[B70] Savriama Y. Gerber S. Baiocco M. Debat V. Fusco G. (2017). Development and evolution of segmentation assessed by geometric morphometrics: the centipede *Strigamia maritima* as a case study. *Arthropod Struct. Dev.* 46 419–428. 10.1016/j.asd.2017.03.002 28302585

[B71] Savriama Y. Gómez J. M. Perfectti F. Klingenberg C. P. (2012). Geometric morphometrics of corolla shape: dissecting components of symmetric and asymmetric variation in *Erysimum mediohispanicum* (Brassicaceae). *New Phytol.* 196 945–954. 10.1111/j.1469-8137.2012.04312.x 22988918

[B72] Savriama Y. Klingenberg C. P. (2011). Beyond bilateral symmetry: geometric morphometric methods for any type of symmetry. *BMC Evol. Biol.* 11:280. 10.1186/1471-2148-11-280 21958045 PMC3209460

[B73] Savriama Y. Neustupa J. Klingenberg C. P. (2010). Geometric morphometrics of symmetry and allometry in *Micrasterias rotata* (Zygnemophyceae, Viridiplantae). *Nova Hedwigia Suppl.* 136 43–54.

[B74] Savriama Y. Vitulo M. Gerber S. Debat V. Fusco G. (2016). Modularity and developmental stability in segmented animals: variation in translational asymmetry in geophilomorph centipedes. *Dev. Genes Evol.* 226 187–196. 10.1007/s00427-016-0538-3 27038021

[B75] Schlager S. (2017). Morpho and Rvcg–Shape Analysis in R: R-Packages for geometric morphometrics, shape analysis and surface manipulations. *Statist. Shape Deform. Anal.* 44 217–256. 10.1016/B978-0-12-810493-4.00011-0

[B76] Schneider C. A. Rasband W. S. Eliceiri K. W. (2012). NIH Image to ImageJ: 25 years of image analysis. *Nat. Methods* 9 671–675. 10.1038/nmeth.2089 22930834 PMC5554542

[B77] Shipunov A. B. Bateman R. M. (2005). Geometric morphometrics as a tool for understanding *Dactylorhiza* (Orchidaceae) diversity in European Russia. *Biol. J. Linn. Soc.* 85 1–12. 10.1111/j.1095-8312.2005.00468.x

[B78] Slice D. Bookstein F. Marcus L. Rohlf F. (1996). Appendix I: a glossary for geometric morphometrics. *Nato Asi Ser. A Life Sci.* 284 531–552.

[B79] Spencer V. Kim M. (2018). Re“CYC”ling molecular regulators in the evolution and development of flower symmetry. *Semin. Cell Dev. Biol.* 79 16–26. 10.1016/j.semcdb.2017.08.052 28864346

[B80] Strelin M. M. Benitez-Vieyra S. Fornoni J. Klingenberg C. P. Cocucci A. (2018). The evolution of floral ontogenetic allometry in the Andean genus *Caiophora* (Loasaceae, subfam. Loasoideae). *Evol. Dev.* 20 29–39. 10.1111/ede.12246 29243890

[B81] Tucić B. Budečević S. Manitašević Jovanović S. Vuleta A. Klingenberg C. P. (2018). Phenotypic plasticity in response to environmental heterogeneity contributes to fluctuating asymmetry in plants: first empirical evidence. *J. Evol. Biol.* 31 197–210. 10.1111/jeb.13207 29134739

[B82] Vujić V. Avramov S. Tarasjev A. Barišić Klisarić N. Živković U. Miljković D. (2015). The effects of traffic- related air pollution on the flower morphology of Iris pumila -comparison of a polluted city area and the unpolluted Deliblato Sands (nature reserve). *Appl. Ecol. Environ. Res.* 13 405–415.

[B83] Wang C.-N. Hsu H.-C. Wang C.-C. Lee T.-K. Kuo Y.-F. (2015). Quantifying floral shape variation in 3D using microcomputed tomography: a case study of a hybrid line between actinomorphic and zygomorphic flowers. *Front. Plant Sci.* 6:724. 10.3389/fpls.2015.00724 26442038 PMC4564768

[B84] Wang P. Liao H. Zhang W. Yu X. Zhang R. Shan H. (2015). Flexibility in the structure of spiral flowers and its underlying mechanisms. *Nat. Plants* 2:15188. 10.1038/nplants.2015.188 27250746

[B85] Weyl H. (1952). *Symmetry.* Princeton, NJ: Princeton University Press 10.1515/9781400874347

