# Supplementary material for: A Step-by-Step Guide for Geometric Morphometrics of Floral Symmetry
Source: Front Plant Sci. 2018 Oct 10;9:1433. doi: 10.3389/fpls.2018.01433 (PMC6191499; doi:10.3389/fpls.2018.01433)
Supplement: Supplementary file 1 [file Data_Sheet_1.ZIP › SupplementaryInformation/CaseStudy1_BilateralSymmetry_Fedia/RscriptBilateralSymmetryFediaProject.html]

Case study 1 Geometric morphometrics of bilaterally symmetric (zygomorphic) flowers (Fedia graciliflora, Valerianaceae) A step-by-step guide using R function C1v and MorphoJ


# Case study 1 Geometric morphometrics of bilaterally symmetric (**zygomorphic**) flowers (*Fedia graciliflora*, Valerianaceae) A step-by-step guide using R function *C1v* and MorphoJ

#### *Yoland SAVRIAMA*

#### *May 4, 2018*

### Overview and software installation

Shape analysis of flowers with bilateral symmetry is already implemented in MorphoJ (see main text, section 4.1.2). However, this software automatically separates the component of symmetric variation from the component of asymmetry, which means that when one runs a PCA on either component one cannot quantify how much variance is accounted by these components relative to the total amount of shape variation. Alternatively, this is feasible using the R function *C1v* as it is explained here.

This is a tutorial that accompanies the supplementary information for the manuscript: *A step-by-step guide for geometric morphometrics of floral symmetry* and describes the procedure to analyze bilaterally symmetric (zygomorphic) flowers with the method of object symmetry (Mardia et al., 2000; Klingenberg et al., 2002; Savriama and Klingenberg, 2011) using the freely available multi-platform R software (R, 2017) that can be downloaded in the following link and must be installed prior to running this code: https://www.r-project.org. An introduction to R and how to use it can be found in this link: https://cran.r-project.org/doc/contrib/Paradis-rdebuts\_en.pdf.

### Preparing the files

Make sure that the files: ***SymmetryFunctions.R***, ***FediaProjectCompiled.TPS***, and ***coFed.txt*** are all saved in the same folder. This folder is known as the current working directory in R and should be specified as explained below. In R, the *#* symbol means that the command line is not run and serves as a comment. The command lines that are readable by R are included in code blocks throughout this tutorial.

R code by Dr. Yoland Savriama (yoland.savriama@helsinki.fi) with the use of *C1v* function written by Dr. Sylvain Gerber (sylvain.gerber@mnhn.fr)

### Loading libraries and source code in R

Installing and loading packages needed in this tutorial:

```
install.packages("geomorph") # an internet connection is needed
library(geomorph) # more details about this package can be found here: https://cran.r-project.org/web/packages/geomorph/index.html
```

The folder which contains all files that is known as the working directory in R needs to be specified by using the following command. Alternatively, one can set it by accessing the appropriate menu in the R Graphical User Interface (GUI) depending on the Operating System used.

Setting up the working directory that contains all files:

```
# wd <- c("~/Mydata/CaseStudy/") # example that needs to be modified according to your own settings
# setwd(wd) #specifying the working directory
```

Loading the necessary functions needed in this tutorial:

```
source("SymmetryFunctions.R") # here, I had already specified that I will be working in a specific directory and I only need to enter the name of the file
```

### Loading data

In this section, I describe the procedure to import the original configurations of landmarks and vectors of relabelling to generate the associated reflected relabelled copies according to the method of object symmetry.

If one has collected landmark data with tpsDig2 or any other software (or function) that outputs the raw coordinates as the .TPS file format, these files can be imported in R via function *readland.tps* as part of the *geomorph* package. Thereafter, the imported data will be converted into a regular 2D data matrix via function *two.d.array* to comply with the formatting requirements of function *C1v*.

Alternatively, one can import a regular text tab delimited file or else that contains the identifiers for the specimens in the first column and landmark coordinates arranged as a 2D data matrix according to this sequence: x1, y1, x2, y2,…, xn, yn or x1, y1, z1, x2, y2, z2,…, xn, yn, zn respectively for 2D and 3D data (e.g. format equivalent to raw landmark coordinates exported from MorphoJ). In this case, landmark coordinates need to be selected and converted as matrix as follows (assuming the first column contains the identifiers for the specimens):

```
# importdta = read.table("example.txt", header = T or F, sep="\t") # header specification depends whether or not original data matrix contains column headers and the type of separator used in the imported dataset should be specified as well (e.g., text tab delimited, comma, comma separated-value, etc.).
```

```
# dta <- as.matrix(importdta[,2:ncol(importdta)]) # select landmark coordinates only and convert them as matrix data assuming that the first column contains non-landmark data (e.g. classifiers, IDs for specimens)
```

Since we are working with landmark data generated by tpsDIG2, we will continue working with this format for the rest of this tutorial. The command line below imports all configurations of landmarks digitized from all series of pictures compiled as a single .TPS file and extracts names of specimens from line *ID=*. Landmark coordinates are multiplied by their scale factor if this is provided for all specimens. If one or more specimens are missing the scale factor, landmarks are treated in their original units.

```
FediaData=readland.tps("FediaProjectCompiled.TPS", specID = "imageID")
```

The next step is to convert the above 3D array into a 2D matrix:

```
FediaRaw = two.d.array(FediaData)
```

### Importing column vector containing information related to relabelling of landmarks

Now, we import the single column vector containing the information about the reflected relabelled landmarks:

```
coFed <- as.matrix(read.table("coFed.txt", header = F, sep="\t")) # text tab delimited file with no column header
```

### Generating reflected relabelled configurations of landmarks

The next step is to generate data with the reflected relabelled copies of the original configurations of landmarks. Copies of the original configuration of landmarks are generated and added right after the matrix containing the original ones, then these copies are reflected with an appropriate relabelling of the landmarks, which swaps the labels of the landmarks that were mirror images of each other with respect to the axis of bilateral symmetry while not affecting the landmarks placed onto this axis since they were mapped onto themselves (object symmetry).

In this case study, we only need function *C1v*, which must have been already copied/imported in R at this point. Below, we apply function *C1v* to our data:

```
RefRelabFedia <- C1v(FediaRaw,coFed,2) #data are in 2D, hence *2* in the third argument
```

### Exporting matrix of all original and transformed relabelled configurations of landmarks for analysis in MorphoJ

The previously generated matrix contains all original ("\_ori“) and reflected relabelled copies (”\_ref"). The following last command line exports this matrix according to a format that is ready to be analyzed in MorphoJ (guide can be accessed here: http://www.flywings.org.uk/MorphoJ\_guide/frameset.htm?index.htm)

```
write.table(RefRelabFedia,file="RefRelabFedia.txt",col.names = F,  quote=F, sep="\t")
```

### Shape analysis of all original and transformed relabelled configurations of landmarks with MorphoJ

Here is a summary of the procedure for Procrustes superimposition (GPA), PCA, and measurement error.

#### *GPA*

- Load the *RefRelabFedia.txt* file in MorphoJ via *File, New Project*, name the project *RefRelabFedia.txt* or else, then select *File, Create New Dataset*. This prompts a window to open in which the user selects the dimensionality of the data (select *2 dimensions*), whether or not the data contains object symmetry (select *no*), select the dataset (*RefRelabFedia.txt*) and name it, and select the file type *text*, the name of the selected file appears in the field *File*. Click *Create Dataset*. The dataset is created and automatically contains all original configurations of landmarks with their reflected and appropriately relabelled copies.
- Create classifiers with *Preliminaries* and choose *Extract new classifier from ID strings*. Create the classifiers for the *individual*, *Reflection*, *Imaging*, and *Digitizing*. Enter a name for a given classifier first, then select the string of characters that correspond to its length. For instance, the classifier for *Individual* extracted from the identifier *Fedia01a1b1\_ori* should only comprise characters between the first and seventh digit *Fedia01*. This classifier can be extracted by entering *1* inside the field for the first character and *7* inside the field for the last character (reading from left to right). Similarly, use *13* and *15* for the classifier *Reflection*, *8* and *9* for the classifier *Imaging*, and *10* and *11* for the classifier *Digitizing*. Alternatively, one can also import predefined classifiers as a separate file (see the MorphoJ on-line documentation for further details, http://www.flywings.org.uk/MorphoJ\_guide/frameset.htm?index.htm).
- To perform a GPA, click *Preliminaries* and select *New Procrustes Fit*. This prompts a window asking how the data should be presented. Select the default choice *align by principal axes* or select another type of alignment if desired and click *Perform Procrustes fit*. A new tab appears representing the coordinates for the consensus (large blue dots) and the deviation around it that is symbolized by the superimposed configurations (small blue dots).

#### *PCA*

- Click on the dataset *RefRelabFedia.txt*. Then go to *Preliminaries* and select *Generate Covariance Matrix*. Select the dataset *RefRelabFedia.txt* in the upper left corner field as well as the data type *Procrustes Coordinates*. Click *Execute* without ticking the box *Pooled within-group covariances*. This generates the corresponding covariance matrices for the data.
- To visualize patterns of variation via PCA, click on the covariance matrix previously created and select *Principal Component Analysis* in the *Variation* menu. This creates a *Graphics* tab with three subtabs: *PC shape changes* that gives the patterns of shape variation for every PC (a right click on this tab gives access to several graphical options), *Eigenvalues* (amount of variance explained by each PC) and *PC scores* (visualization of individuals in the shape space). A *Results* tab is also produced that reports the results from the PCA (i.e. eigenvalues and eigenvectors also known as principal components coefficients). If an outline has been already imported inside MorphoJ or if a wireframe graph is available, the investigator can also select either type of visualization as well by right-clicking inside the *PC shape changes* of the *Graphics* tab and select *Change the type of graph* (see the MorphoJ on-line documentation for further details about the creation of wireframes and outlines, http://www.flywings.org.uk/MorphoJ\_guide/frameset.htm?index.htm).
- If the investigator is only interested in carrying analyses on the component of symmetric variation (i.e. variation among flowers) and wants to discard the asymmetry, one can calculate it by simply clicking on *Preliminaries* and selecting *Average Observations By*, then pick the *RefRelabFedia.txt* dataset, select *Average by* and choose the identifiers corresponding to *Individual*, with *all Data types* remaining selected. Click execute. This creates a new dataset with the same name as the original dataset with the extension *averaged* added to it. To visualize the patterns of morphological variation associated with this component, simply select this newly created dataset and go to *Preliminaries* then *Generate Covariance Matrix*, then go to *Variation* and choose *Principal Component Analysis*.

#### *Measurement Error*

- A one way Procrutes ANOVA to test for measurement error due to imaging and digitizing is implemented inside MorphoJ. Select *File, Create New Dataset*, select *2 dimensions* and for object symmetry (select *no*), name the dataset (*FediaOriginal*), and select the file type *NTSYSpc*, the name of the selected file appears inside the field *File*. Click *Create Dataset*. The dataset is created and contains all original configurations of landmarks only.
- Click on the newly generated dataset *FediaOriginal* then select *Variation* and go to *Procrustes ANOVA*. This will prompt a new window in which you can name the analysis and enter the main effects for your design that correspond to the classifiers previously created. Enter the classifier corresponding to *Individual*, Error 1 is the *Imaging* classifier and Error 2 is the *Digitizing* classifier. Click *Execute*. This will create a results tab and covariance matrices for each effect in the project tree. Select all the covariance matrices (hold shift + left click). Go to the *Variation* menu and select *Principal Component Analysis* to run a PCA on each of the previously selected covariance matrices to visualize the corresponding patterns of shape changes for each effect.

### References

Klingenberg, C.P., Barluenga, M., and Meyer, A. (2002). Shape analysis of symmetric structures: quantifying variation among individuals and asymmetry. Evolution 56, 1909-1920.   
 Mardia, K.V., Bookstein, F.L., and Moreton, I.J. (2000). Statistical assessment of bilateral symmetry of shapes. Biometrika, 285-300.   
 R (2017). R: A language and environment for statistical computing. R Foundation for Statistical Computing.   
 Savriama, Y., and Klingenberg, C.P. (2011). Beyond bilateral symmetry: geometric morphometric methods for any type of symmetry. BMC Evolutionary Biology 11, 1.
