# Supplementary material for: A Step-by-Step Guide for Geometric Morphometrics of Floral Symmetry
Source: Front Plant Sci. 2018 Oct 10;9:1433. doi: 10.3389/fpls.2018.01433 (PMC6191499; doi:10.3389/fpls.2018.01433)
Supplement: Supplementary file 1 [file Data_Sheet_1.ZIP › SupplementaryInformation/CaseStudy2_TwoPerpendicularAxesBilateralSymmetry_Erysimum/RscriptDisymmetryErysimumProject.html]

Case study 2 Geometric morphometrics of flowers (Erysimum mediohispanicum, Brassicaceae) with two perpendicular axes of symmetry (disymmetric or bisymmetric) A step-by-step guide using R function C1v and MorphoJ


# Case study 2 Geometric morphometrics of flowers (*Erysimum mediohispanicum*, Brassicaceae) with two perpendicular axes of symmetry (**disymmetric or bisymmetric**) A step-by-step guide using R function *C1v* and MorphoJ

#### *Yoland SAVRIAMA*

#### *May 4, 2018*

### Overview and software installation

This is a tutorial that accompanies the supplementary information for the manuscript: *A step-by-step guide for geometric morphometrics of floral symmetry* and describes the procedure to analyze complex symmetric (disymmetric or bisymmetric) flowers with the method of object symmetry (Mardia et al., 2000; Klingenberg et al., 2002; Savriama and Klingenberg, 2011) using the freely available multi-platform R software (R, 2017) that can be downloaded in the following link and must be installed prior to running this code: https://www.r-project.org. An introduction to R and how to use it can be found in this link: https://cran.r-project.org/doc/contrib/Paradis-rdebuts\_en.pdf.

### Preparing the files

Make sure that the files: ***SymmetryFunctions.R***, ***ErysimumRawData.TPS***, ***coEryLR.txt***, ***coEryAdAb.txt***, and ***coEry.txt*** are all saved in the same folder. This folder is known as the current working directory in R and should be specified as explained below. In R, the *#* symbol means that the command line is not run and serves as a comment. The command lines that are readable by R are included in code blocks throughout this tutorial.

R code by Dr. Yoland Savriama (yoland.savriama@helsinki.fi) with the use of *C1v* function written by Dr. Sylvain Gerber (sylvain.gerber@mnhn.fr)

### Loading libraries and source code in R

Installing and loading packages needed in this tutorial:

```
install.packages("geomorph") # an internet connection is needed
library(geomorph) # more details about this package can be found here: https://cran.r-project.org/web/packages/geomorph/index.html
```

The folder which contains all files that is known as the working directory in R needs to be specified by using the following command. Alternatively, one can set it by accessing the appropriate menu in the R Graphical User Interface (GUI) depending on the Operating System used.

Setting up the working directory that contains all files:

```
# wd <- c("~/Mydata/CaseStudy/") # example that needs to be modified according to your own settings
# setwd(wd) #specifying the working directory
```

Loading the necessary functions needed in this tutorial:

```
source("SymmetryFunctions.R") # here, I had already specified that I will be working in a specific directory and I only need to enter the name of the file
```

### Loading data

In this section, I describe the procedure to import the original configurations of landmarks and vectors of relabelling to generate the associated transformed relabelled copies according to the method of object symmetry.

If one has collected landmark data with tpsDig2 or any other software (or function) that outputs the raw coordinates as the .TPS file format, these files can be imported in R via function *readland.tps* as part of the *geomorph* package. Thereafter, the imported data will be converted into a regular 2D data matrix via function *two.d.array* to comply with the formatting requirements of function *C1v* and *Cn*.

Alternatively, one can import a regular text tab delimited file or else that contains the identifiers for the specimens in the first column and landmark coordinates arranged as a 2D data matrix according to this sequence: x1, y1, x2, y2,…, xn, yn or x1, y1, z1, x2, y2, z2,…, xn, yn, zn respectively for 2D and 3D data (e.g. format equivalent to raw landmark coordinates exported from MorphoJ). In this case, landmark coordinates need to be selected and converted as matrix as follows (assuming the first column contains the identifiers for the specimens):

```
# importdta = read.table("example.txt", header = T or F, sep="\t") # header specification depends whether or not original data matrix contains column headers and the type of separator used in the imported dataset should be specified as well (e.g., text tab delimited, comma, comma separated-value, etc.).
```

```
# dta <- as.matrix(importdta[,2:ncol(importdta)]) # select landmark coordinates only and convert them as matrix data assuming that the first column contains non-landmark data (e.g. classifiers, IDs for specimens)
```

Since we are working with landmark data generated by tpsDIG2, we will continue working with this format for the rest of this tutorial. The command line below imports all configurations of landmarks digitized from all series of pictures compiled as a single .TPS file and extracts names of specimens from line *ID=*. Landmark coordinates are multiplied by their scale factor if this is provided for all specimens. If one or more specimens are missing the scale factor, landmarks are treated in their original units.

```
EryData=readland.tps("ErysimumRawData.tps", specID = "imageID")
```

The next step is to convert the above 3D array into a 2D matrix:

```
EryRaw = two.d.array(EryData)
```

As mentioned in the main text (section 4.2.2), there are two possibilities to treat this case study either by considering it as with two perpendicular axes of bilateral symmetry (Solution 1) or with combination of bilateral symmetry and rotational symmetry of order 2 (by 180 degrees) (Solution 2). These procedures are mutually equivalent and generate the same results.

#### Solution 1 Two perpendicular axes of symmetry (vertical left-right and horizontal adaxial-abaxial) using function *C1v* applied twice successively

- Importing column vector containing information related to relabelling of landmarks

The *coEryLR.txt* file or object containing information about the relabelling of landmarks for reflection about the vertical left-right axis is imported. The reflected relabelled copies about the vertical left-right axis are generated first using function *C1v*.

```
coEryLR <- as.matrix(read.table("coEryLR.txt", header = F, sep="\t")) #text tab delimited file with no column header
```

- Generating transformed relabelled configurations of landmarks

The next step is to generate data with the reflected relabelled copies of the original configurations of landmarks about the vertical left-right axis of symmetry. Copies of the original configuration of landmarks are generated and added right after the matrix containing the original ones, then these copies are reflected with an appropriate relabelling of the landmarks, which swaps the labels of the landmarks that were mirror images of each other with respect to the axis of bilateral symmetry while not affecting the landmarks placed onto this axis since they were mapped onto themselves (object symmetry). In this case study, we use function *C1v*, which must have been already copied/imported in R at this point. Below, we apply function *C1v* to our data:

```
RefRelabEry1_2 <- C1v(EryRaw,coEryLR,2) #using the vector of relabelling and applying function C1v to obtain all reflected relabelled copies about the vertical left-right axis of bilateral symmetry. Data are in 2D, hence 2 in the third argument
```

Then, the *coEryAdAb.txt* file or object containing information about the relabelling of landmarks for reflection about the horizontal adaxial-abaxial axis is imported. The reflected relabelled copies about the horizontal adaxial-abaxial axis as well as the transformed relabelled copies following two successive reflections (equivalent to rotation by 180 degrees) are generated using the function *C1v* a second time on the data previously generated.

```
coEryAdAb <- as.matrix(read.table("coEryAdAb.txt", header = F, sep="\t")) #text tab delimited file with no column header
RefRelabEry2_2 <- C1v(RefRelabEry1_2,coEryAdAb,2) # using the vector of relabelling and applying function C1v to obtain all reflected relabelled copies about the horizontal adaxial-abaxial axis of bilateral symmetry, but also generating transformed copies that are combinations of two successive reflections (i.e. rotation of order 2 or by 180 degrees). Data are in 2D, hence 2 in the third argument
```

Finally, the user can directly go to the section *Exporting matrix of all original and transformed relabelled configurations of landmarks to export data for shape analysis in MorphoJ*.

#### Solution 2 Bilateral symmetry combined with rotational symmetry of order 2 (by 180 degrees) using functions *C1v* and *Cn* applied successively

- Importing matrix containing information related to relabelling of landmarks

Here, we import the matrix containing the information about relabelled landmarks according to reflection and rotation of order 2 (by 180 degrees). In this case study, the first column should contain the information about the relabeling for reflection and the second (last) indicates the relabeling for rotation by 180 degrees.

```
coEry <- as.matrix(read.table("coEry.txt", header = F, sep="\t")) # text tab delimited file with no column header
```

- Generating transformed relabelled configurations of landmarks

The next step is to generate data with the transformed relabelled copies of the original configurations of landmarks. Copies of the original configurations of landmarks are generated first, then their reflected relabelled copies, followed by their rotated relabelled copies (equivalent to two successive reflections about both axes), and finally by their reflected rotated relabelled copies (equivalent to reflection about the adaxial-abaxial axis). In this case study, we first need function *C1v* to generate the reflected relabelled copies, which must have been already copied/imported in R at this point. Below, we apply function *C1v* to our data:

```
RefRelabEry <- C1v(EryRaw,coEry[,1],2) #using the vector of relabelling and applying function C1v to obtain all reflected relabelled copies. The appropriate corresponding vector column is subtracted/selected from the original matrix to match the second argument of the function. Data are in 2D, hence 2 in the third argument
```

Finally, we need function *Cn* to generate the rotated relabelled copies as well as the reflected rotated relabelled copies by combining it with the reflected relabelled data previously generated by function *C1v*. This is necessary to obtain all transformed relabelled copies corresponding to the symmetry group of the flower under study. Function *Cn* must have been already copied/imported in R at this point. Below, we apply function *Cn* to our data:

```
RefRotRelabEry = Cn(RefRelabEry, coEry[,2],2)
# vector of relabelling for rotation by 180 degrees is extracted from the second column of the coEry matrix. Data are in 2D, hence *2* in the third argument
```

### Exporting matrix of all original and transformed relabelled configurations of landmarks for analysis in MorphoJ

#### Solution 1 Two perpendicular axes of symmetry (vertical left-right and horizontal adaxial-abaxial)

The previously generated matrix contains all original (*ori\_ori*), reflected relabelled copy about the vertical left-right axis only (*ref\_ori*), reflected relabelled copy about the horizontal adaxial-abaxial axis only (*ori\_ref*), and reflected relabelled copy about both perpendicular axes or equivalently as rotation of order 2 (by 180 degrees in this example) (*ref\_ref*). The following last command line exports this matrix according to a format that is ready to be analyzed in MorphoJ (guide can be accessed here: http://www.flywings.org.uk/MorphoJ\_guide/frameset.htm?index.htm)

```
write.table(RefRelabEry2_2,file="EryRefRefRelab.txt",col.names = F,  quote=F, sep="\t")
```

#### Solution 2 Bilateral symmetry combined with rotational symmetry of order 2 (by 180 degrees)

The previously generated matrix contains all original (*ori\_Cn00*), reflected relabelled copies (*ref\_Cn00*), rotated relabelled copies by 180 degrees (*ori\_Cn01*), and reflected rotated relabelled copies by 180 degrees (*ref\_Cn01*). The following last command line exports this matrix according to a format that is ready to be analyzed in MorphoJ (guide can be accessed here: http://www.flywings.org.uk/MorphoJ\_guide/frameset.htm?index.htm)

```
write.table(RefRotRelabEry,file="EryRefRotRelab.txt",col.names = F,  quote=F, sep="\t")
```

### Shape analysis of all original and transformed relabelled configurations of landmarks with MorphoJ

Here is a summary of the procedure for Procrustes superimposition (GPA) and PCA. Pictures nor landmarks were taken twice, hence measurement error could not be assessed here (see sections 3.2 and 3.3 and Supplementary Material for case study 1 for the detailed procedure to estimate measurement error in MorphoJ).

#### *GPA*

- Load the *EryRefRefRelab.txt* or *EryRefRotRelab.txt* file in MorphoJ via *File, New Project*, name the project *EryRefRefRelab.txt* or *EryRefRotRelab.txt* or else, then select *File, Create New Dataset*. This prompts a window to open in which the user selects the dimensionality of the data (select *2 dimensions*), whether or not the data contains object symmetry (select *no*), select the dataset *EryRefRefRelab.txt* or *EryRefRotRelab.txt* and name it, and select the file type *text*, the name of the selected file appears in the field *File*. Click *Create Dataset*. The dataset is created and automatically contains all original configurations of landmarks with their reflected and appropriately relabelled copies.
- Create classifiers with *Preliminaries* and choose *Extract new classifier from ID strings*. Create the classifiers for the *individual* and *Transformation*. Enter a name for a given classifier first, then select the string of characters that correspond to its length. For instance, the classifier for *Individual* extracted from the identifier *001\_ori\_ori* should only comprise characters between the first and third digit *001*. This classifier can be extracted by entering *1* in the field for the first character and *3* in the field for the last character (reading from left to right). Similarly, use *6* and *-1* for the classifier *Transformation*. Alternatively, one can also import predefined classifiers as a separate file (see the MorphoJ on-line documentation for further details, http://www.flywings.org.uk/MorphoJ\_guide/frameset.htm?index.htm).
- To perform a GPA, click *Preliminaries* and select *New Procrustes Fit*. This prompts a window asking how the data should be presented. Select the default choice *align by principal axes* or select another type of alignment if desired and click *Perform Procrustes fit*. A new tab appears representing the coordinates for the consensus (large blue dots) and the deviation around it that is symbolized by the superimposed configurations (small blue dots).

#### *PCA*

- Click on the dataset *EryRefRotRelab.txt*. Then go to *Preliminaries* and select *Generate Covariance Matrix*. Select the dataset *EryRefRotRelab.txt* in the upper left corner field as well as the data type *Procrustes Coordinates*. Click *Execute* without ticking the box *Pooled within-group covariances*. This generates the corresponding covariance matrices for the data.
- To visualize patterns of variation via PCA, click on the covariance matrix previously created and select *Principal Component Analysis* in the *Variation* menu. This creates a *Graphics* tab with three subtabs: *PC shape changes* that gives the patterns of shape variation for every PC (a right click on this tab gives access to several graphical options), *Eigenvalues* (amount of variance explained by each PC) and *PC scores* (visualization of individuals in the shape space). A *Results* tab is also produced that reports the results from the PCA (i.e. eigenvalues and eigenvectors also known as principal components coefficients). If an outline has been already imported inside MorphoJ or if a wireframe graph is available, the investigator can also select either type of visualization as well by right-clicking inside the *PC shape changes* of the *Graphics* tab and select *Change the type of graph* (see the MorphoJ on-line documentation for further details about the creation of wireframes and outlines, http://www.flywings.org.uk/MorphoJ\_guide/frameset.htm?index.htm).
- If the investigator is only interested in carrying analyses on the component of symmetric variation (i.e. variation among flowers) and wants to discard the asymmetry, one can calculate it by simply clicking on *Preliminaries* and selecting *Average Observations By*, then pick the *EryRefRefRelab.txt* or *EryRefRotRelab.txt* dataset, select *Average by* and choose the identifiers corresponding to *Individual*, with *all Data types* remaining selected. Click execute. This creates a new dataset with the same name as the original dataset with the extension *averaged* added to it. To visualize the patterns of morphological variation associated with this component, simply select this newly created dataset and go to *Preliminaries* then *Generate Covariance Matrix*, then go to *Variation* and choose *Principal Component Analysis*.

### References

Klingenberg, C.P., Barluenga, M., and Meyer, A. (2002). Shape analysis of symmetric structures: quantifying variation among individuals and asymmetry. Evolution 56, 1909-1920.   
 Mardia, K.V., Bookstein, F.L., and Moreton, I.J. (2000). Statistical assessment of bilateral symmetry of shapes. Biometrika, 285-300.   
 R (2017). R: A language and environment for statistical computing. R Foundation for Statistical Computing.   
 Savriama, Y., and Klingenberg, C.P. (2011). Beyond bilateral symmetry: geometric morphometric methods for any type of symmetry. BMC Evolutionary Biology 11, 1.
